# Identification of YL064 as a novel inhibitor of ATXN3 to suppress tumor growth and metastasis by promoting degradation of oncogenic substrates

**DOI:** 10.1038/s41419-026-09171-5

**Published:** 2026-08-06

**Authors:** Zhenge Zhang, Wenhui Bai, Yayue Tan, Guanyi Li, Youping Zhang, Wenxuan Wu, Chujiao Zhu, Xingyu Liu, Manyu Zhang, Hu Lei, Hanzhang Xu, Li Yang, Ligen Liu, Yaxue Zhao, Zhujun Yao, Qi Zhu, Yingying Wang, Yingli Wu

**Affiliations:** 1 Department of Hematology, Tongren Hospital & Department of Pathophysiology, Key Laboratory of Cell Differentiation and Apoptosis of the National Ministry of Education, Shanghai Jiao Tong University School of Medicine, Shanghai, China; 2 School of Pharmaceutical Sciences, Shanghai Jiao Tong University, Shanghai, China; 3 State Key Laboratory of Coordination Chemistry and Jiangsu Key Laboratory of Advanced Organic Materials, School of Chemistry and Chemical Engineering, Nanjing University, Nanjing, China; 4 Department of Oral and Maxillofacial-Head and Neck Oncology, Ninth People’s Hospital, College of Stomatology, Shanghai Jiao Tong University School of Medicine, Shanghai, China

**Keywords:** High-throughput screening, Breast cancer

## Abstract

ATXN3 has recently emerged as a pro-oncogenic deubiquitinating enzyme (DUB) involved in the progression of multiple cancers, including breast and prostate cancer. However, the therapeutic potential of targeting ATXN3 remains largely unexplored due to the lack of effective inhibitors. In this study, we performed an Ub-AMC-based high-throughput screening assay using full-length ATXN3 and identified YL064, a derivative of the natural product sinomenine isolated from *Sinomenium acutum*, as a novel ATXN3 inhibitor. Direct interaction between YL064 and ATXN3 was confirmed by thermal shift assay, cellular thermal shift assay, surface plasmon resonance, and molecular docking analysis. Functionally, YL064 suppressed cancer cell proliferation, induced cell-cycle arrest, and reduced metastatic potential. Mechanistically, YL064 inhibited ATXN3 deubiquitinating activity and promoted the ubiquitination and proteasomal degradation of ATXN3 substrates, including KLF4 and YAP. Furthermore, YL064 significantly inhibited tumor growth in an orthotopic breast cancer model in vivo. Collectively, our findings establish YL064 as a promising ATXN3 inhibitor that targets the ubiquitin-proteasome pathway to exert anti-tumor effects, highlighting its potential as a novel therapeutic agent for ATXN3-driven cancers.

**Figure:**
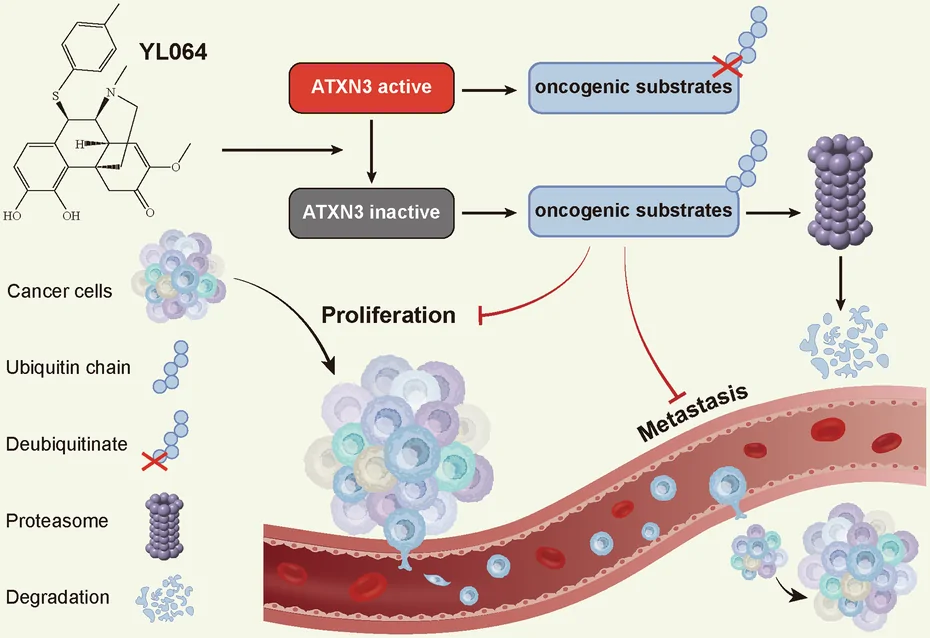

## Introduction

The deubiquitinating enzyme ATXN3 (Ataxin-3), a member of the Machado-Joseph disease (MJD) protease family, is a highly conserved and ubiquitously expressed protein encoded on chromosome 14q32.1 [1, 2]. Initially characterized for its role in spinocerebellar ataxia type 3 (SCA3) due to polyglutamine (polyQ) expansion [3], ATXN3 is now recognized as a multifunctional protein involved in diverse cellular processes, including maintaining genome integrity by regulating various key DNA damage response proteins [4, 5], acting as a transcriptional repressor [6], regulating DNA replication [7], regulating the cytoskeleton [8], apoptosis [8, 9], and cell-cycle progression [5, 10]. Beyond its physiological roles, ATXN3 has garnered increasing attention for its context-dependent functions in cancer. While it may act as a tumor suppressor in certain contexts, such as colorectal cancer [11], accumulating evidence highlights its pro-oncogenic role across various malignancies. Notably, its DUB activity promotes tumor progression by stabilizing key oncogenic substrates: it deubiquitinates and stabilizes Krüppel-like factor 4 (KLF4) in breast cancer [12], inhibits the degradation of Histone Deacetylase 6 (HDAC6) via the ubiquitin-proteasome pathway (UPP) in pancreatic cancer [13], and stabilizes Yes-associated protein (YAP) in prostate cancer [14]. These findings collectively nominate ATXN3 as a promising therapeutic target in these cancers.

Sinomenine, an alkaloid extracted from the medicinal plant *Sinomenium acutum*, is clinically used for treating rheumatoid arthritis [15] and has shown potential anti-tumor activity in preclinical models, such as glioblastoma and colon cancer [16, 17]. Its clinical translation in oncology is hampered by limitations including the requirement for high doses and associated side effects. However, it retains significant development potential owing to its involvement in a wide range of signaling pathways and favorable predictive results for multiple target sites [18]. To overcome these drawbacks, derivative synthesis has emerged as a promising strategy. The derivatives that have undergone structural modifications mainly at the A ring and C ring have shown excellent anti-tumor function [19]. A series of sinomenine derivatives synthesized by our group, undergoing modification on the B ring, have also presented better biological activity. As one of these derivatives, YL064 demonstrated superior therapeutic efficacy, therefore, exploring its functions and therapeutic effects will further promote the research progress of sinomenine and its derivatives in disease treatment.

In this study, we aimed to identify and characterize a novel inhibitor of ATXN3 for cancer treatment. Through screening a series of small molecular and natural compounds, we identified YL064 as a potential inhibitor of ATXN3. Using a combination of biochemical, cellular, and in vivo models, we demonstrated that YL064 exerted potent anti-tumor effects by inhibiting ATXN3’s DUB activity, thereby promoting the degradation of its key oncogenic substrate. Our work thus not only reports YL064 as a promising lead compound but also validates the therapeutic strategy of targeting ATXN3 in tumors.

## Results

### YL064 is a promising inhibitor of ATXN3

**Fig. 1:**
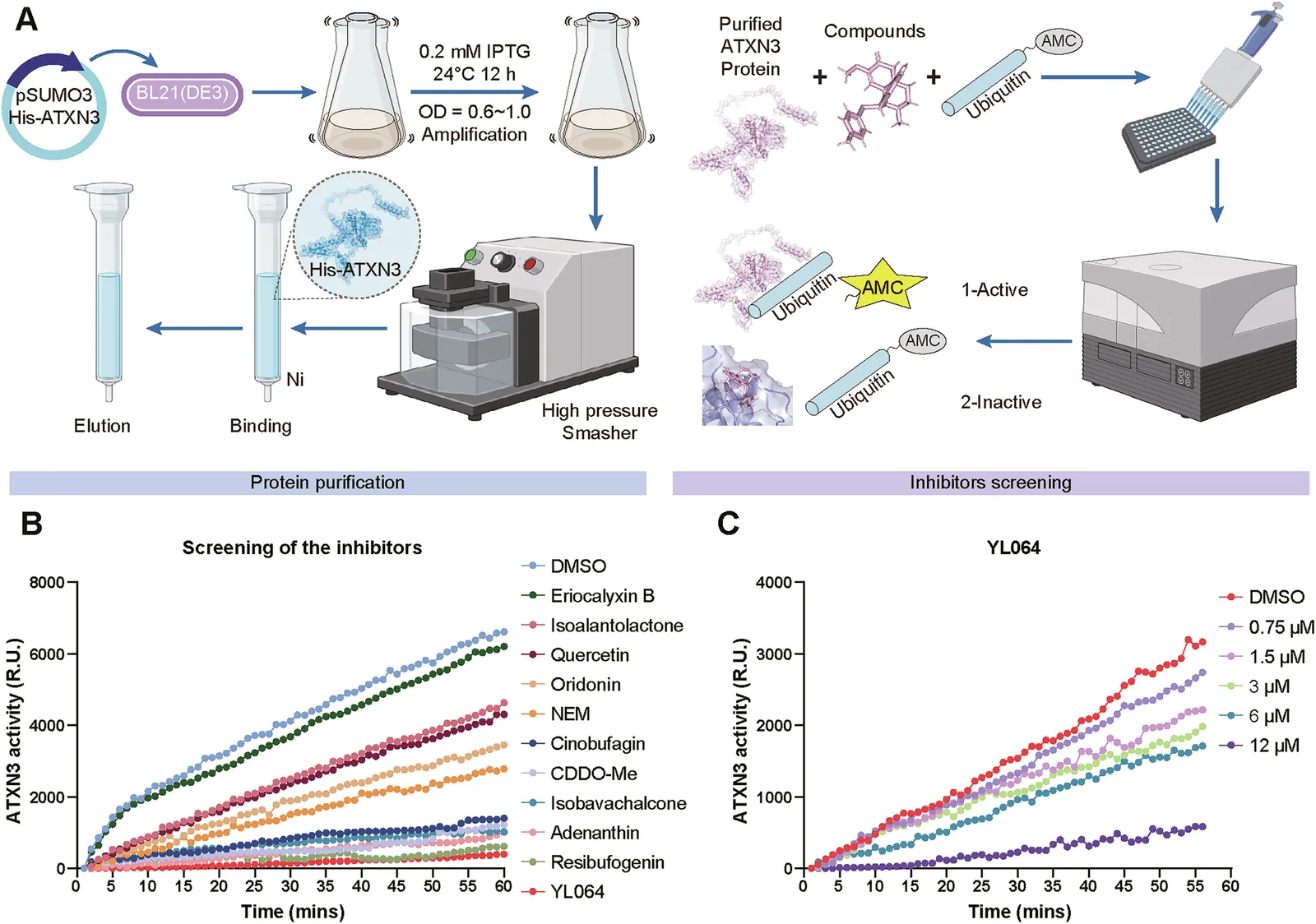
Identification of YL064 as an inhibitor of ATXN3. **A** Schematic illustration of the workflow used to discover ATXN3 inhibitors. Full-length ATXN3 protein was obtained through prokaryotic expression and purification, followed by screening potential inhibitors using the Ub-AMC-based enzymatic assay. Some elements were created with BioRender.com. **B** Enzyme activity profiles of representative inhibitor candidates. Purified ATXN3 was incubated with DMSO, NEM, or various test compounds for 15 min, after which the reaction was initiated by addition of the Ub-AMC substrate. Enzymatic activity was determined by monitoring fluorescence intensity. All compounds were tested at a final concentration of 12 μM. **C** Dose–response relationship of YL064. ATXN3 activity was quantified using the Ub-AMC assay after incubation with gradient concentrations of YL064. (R.U. denotes relative fluorescence units).

To identify potential inhibitors targeting ATXN3, we reconstituted its DUB activity in vitro by purifying the full-length ATXN3 protein. Using the fluorogenic substrate ubiquitin-AMC (Ub-AMC), a compound library of natural products and their analogs, such as bardoxolone methyl (CDDO-Me), oridonin, and isoalantolactone, was screened (Fig. 1A). Through this preliminary screening, several candidates, including YL064, exhibited notable inhibitory effects on ATXN3 activity (Fig. 1B).

To further evaluate the stability and pharmacological potential of these compounds, dose–response assays were conducted for comparative analysis (Fig. 1C & Supplementary Fig. S1). The results revealed that YL064 suppressed ATXN3 enzymatic activity in a concentration-dependent manner and achieved substantial inhibition at relatively low concentrations, indicating both high potency and stable efficacy (Fig. 1C).

### YL064 directly interacts with ATXN3

**Fig. 2:**
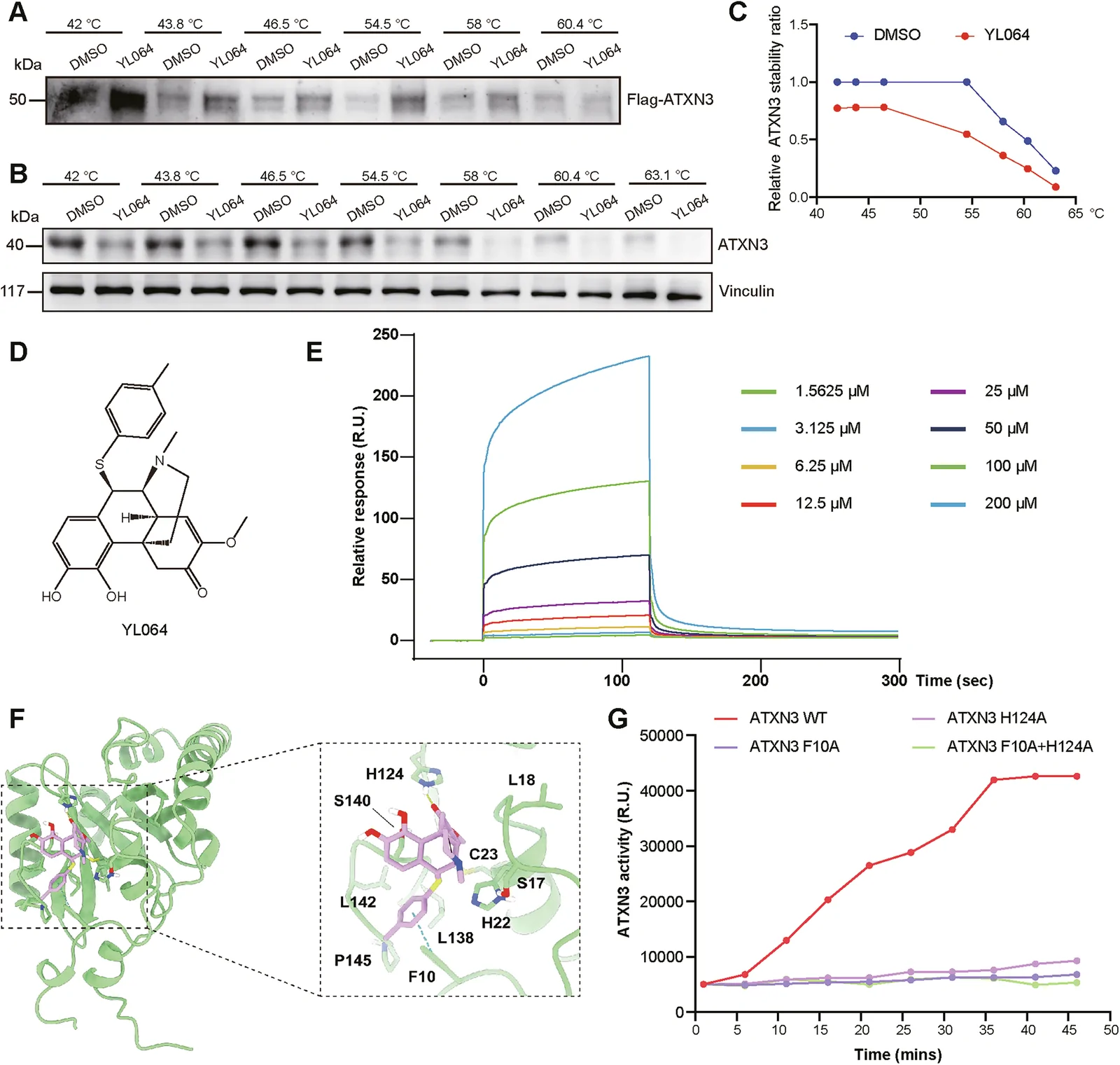
YL064 interacts with ATXN3 directly. **A** The TSA result of the interaction between purified Flag-ATXN3 protein and YL064 (100 μM). **B** The CETSA result of the temperature stage of the interaction between ATXN3 and YL064 (100 μM) in Py8119 cell lines. **C** The relative intensity ratio of ATXN3 stability in (**B**). **D** The structural formula of YL064. **E** The SPR assay demonstrated direct binding between YL064 and ATXN3. The KD value is 649 μM. **F** Docked pose of YL064 (plum sticks) in ATXN3 (light green ribbon and sticks). Hydrogen bonds were depicted as yellow dashed lines, and the π-π interaction was shown as sky blue dashed lines. **G** Based on the docking model in (**F**), the predicted binding residues were mutated, and recombinant ATXN3 proteins (WT, F10A, H124A, and the double mutant F10A/H124A) were purified for Ub-AMC enzymatic analysis. The mutant proteins exhibited impaired deubiquitinating activity. (R.U. denotes relative fluorescence units).

Given that YL064 markedly suppressed ATXN3 enzymatic activity in vitro, we hypothesized that this compound might interact directly with ATXN3. To examine this possibility, we performed Thermal shift assay (TSA) and Cellular thermal shift assay (CETSA). The TSA result provided clear evidence that YL064 binds directly to the purified ATXN3 protein (Fig. 2A). Furthermore, CETSA analyses conducted across both concentration and temperature gradients demonstrated that YL064 decreased the thermal stability of ATXN3 relative to the DMSO control, further supporting a direct interaction between YL064 and ATXN3 within the cellular environment of breast cancer cells (Fig. 2B, C). The direct binding of YL064 (Fig. 2D) to ATXN3 was validated by SPR analysis (Fig. 2E).

To further analyze the binding mechanisms, molecular docking was performed to predict the binding pose of YL064 in ATXN3. In the docked pose, YL064 formed π-π interaction with F10 and a hydrogen bond with H124. The benzyl group inserted into a hydrophobic pocket composed of residues F10, L138, L142, and P145. Additionally, YL064 formed hydrophobic interactions with the side chain methylene groups of S17, H22 and S140 (Fig. 2F).

To determine whether the predicted residues are critical for the interaction between YL064 and ATXN3, site-directed mutagenesis was performed to generate the single mutants F10A and H124A, as well as the double mutant F10A/H124A. Recombinant mutant ATXN3 proteins were subsequently purified and subjected to TSA analysis (Supplementary Fig. S2A, B). The thermal stability of the mutant proteins was reduced by YL064 even more markedly than that of the wild-type (WT) protein, indicating that YL064 was still able to bind ATXN3 despite these mutations (Supplementary Fig. S2A, B). However, this might be due to the changes of the intrinsic stability of the mutant proteins, as TSA results showed that the thermal stability of the mutant ATXN3 proteins was substantially impaired (Supplementary Fig. S2C). Interestingly, compared with wild-type ATXN3, all mutant proteins exhibited markedly reduced deubiquitinating activity, indicating that these residues are critical for maintaining the catalytic function of ATXN3 (Fig. 2G). These findings suggest that F10 and H124 are critical for the catalytic activity of ATXN3 and may also participate in the interaction between ATXN3 and YL064.

### YL064 exhibits greater potency than sinomenine in inhibiting ATXN3

**Fig. 3:**
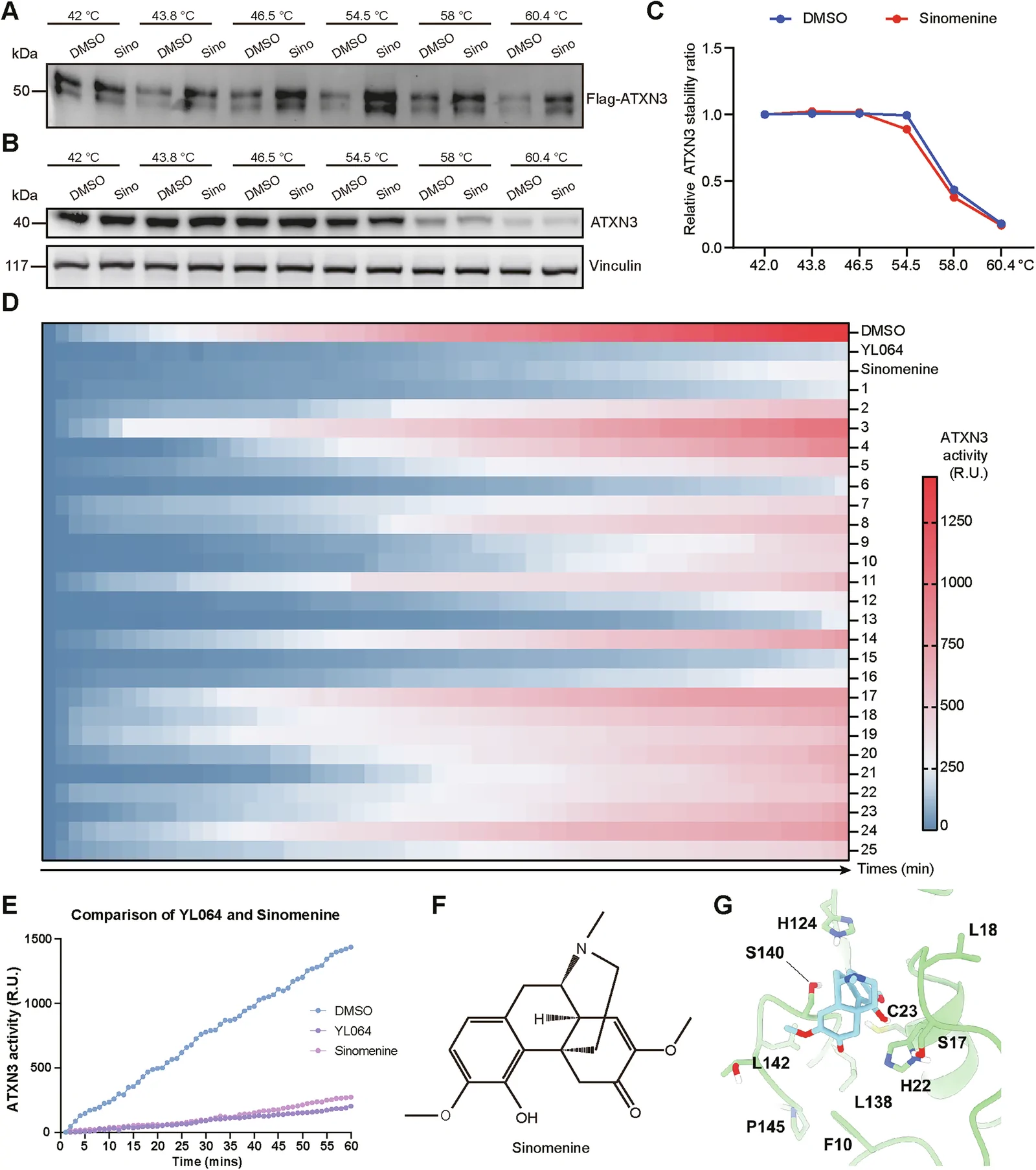
Sinomenine inhibits ATXN3 with lower activity than YL064. **A** The TSA result of the interaction between purified Flag-ATXN3 protein and sinomenine (100 μM). **B** The CETSA result of the temperature stage of the interaction between ATXN3 and sinomenine (100 μM) in Py8119 cell lines. **C** The relative intensity ratio of ATXN3 stability in (**B**). **D** Screening of sinomenine (100 μM) and its derivatives (12 μM). **E** The comparison between sinomenine and YL064 in (**D**). **F** The structural formula of sinomenine. **G** Docked pose of sinomenine (blue sticks) in ATXN3 (light green ribbon and sticks).

As described in the introduction, YL064 is a derivative of sinomenine. To determine whether the inhibitory effect of YL064 originated from its parent compound, we examined the potential interactions between ATXN3 and sinomenine, as well as its derivatives. TSA confirmed that sinomenine was capable of binding to the purified ATXN3 protein (Fig. 3A). In contrast, CETSA result indicated that sinomenine did not alter the thermal stability of ATXN3 at the same concentration used for YL064, suggesting a relatively weak interaction in the cellular context (Fig. 3B, C).

Subsequently, enzymatic activity assays were performed to systematically assess the inhibitory effects of sinomenine and its derivatives. Interestingly, both sinomenine and the majority of its analogs displayed measurable inhibition of ATXN3 (Fig. 3D & Supplementary Table S3). However, when comparing their potencies, sinomenine required substantially higher concentrations to achieve a level of inhibition comparable to that induced by low doses of YL064 (Fig. 3E).

To gain structural insight into their distinct binding mode, we compared the docking conformations of sinomenine (Fig. 3F) and YL064. Unlike YL064, sinomenine exhibited an alternative binding pose that lacked the hydrogen bond and π-π interaction observed with YL064 (Fig. 3G). Additionally, sinomenine was unable to extend into the hydrophobic pocket. These structural differences in binding mode might account for the observed activity differences between YL064 and sinomenine.

### YL064 promotes the degradation of ATXN3 substrate KLF4 in breast cancer through UPP

**Fig. 4:**
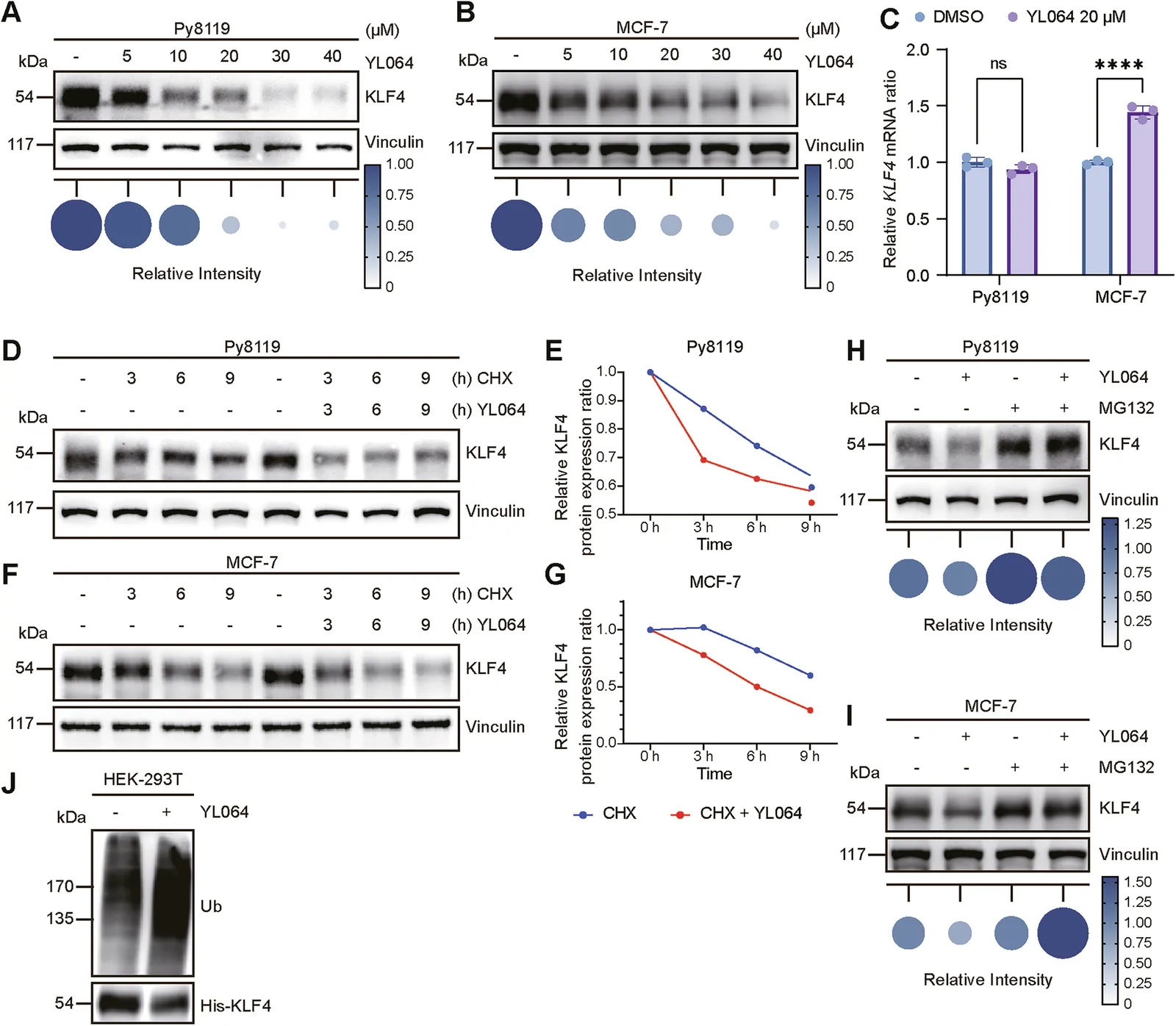
YL064 facilitates KLF4 degradation via UPP. **A**, **B** Western blot analysis showing the concentration-dependent reduction of KLF4 protein in Py8119 and MCF-7 cells following 12 h exposure to YL064, with densitometric normalization to Vinculin. **C** Transcriptional levels of *KLF4* in Py8119 and MCF-7 cells after YL064 treatment. **D**–**G** Determination of KLF4 protein half-life and the corresponding quantification of relative expression ratios. **H**, **I** The proteasome blocker MG132 prevented YL064-induced KLF4 degradation in Py8119 and MCF-7 cells. **J** Analysis of KLF4 ubiquitination in HEK-293T cells transfected with His-KLF4 and treated with 5 μM MG132 for 4 h, with or without subsequent 20 μM YL064 exposure for 12 h. (****P < 0.0001. Data were analyzed by Student’s *t* test).

As ATXN3 is highly expressed in breast and prostate cancers (Supplementary Fig. S3), we next investigated the functional effects and mechanisms of YL064 in breast and prostate cancer cell lines. To determine whether YL064 suppresses the deubiquitinating activity of ATXN3, we examined KLF4, a known substrate of ATXN3 in breast cancer. First, we assessed the effect of YL064 on KLF4 expression across a range of concentrations. The results demonstrated that KLF4 protein levels decreased in a dose-dependent manner following YL064 treatment (Fig. 4A, B). To determine whether this down-regulation occurred at the transcriptional level, RT-qPCR analysis was conducted. The mRNA levels of *KLF4* showed no significant change in either Py8119 or MCF-7 cells (Fig. 4C), indicating that the YL064-induced suppression of KLF4 occurred post-transcriptionally. We further investigated whether YL064 promoted KLF4 degradation. Measurement of KLF4 protein stability revealed that its half-life was shortened upon YL064 exposure (Fig. 4D–G). Treatment with the proteasome inhibitor MG132 effectively restored KLF4 protein levels reduced by YL064 (Fig. 4H, I). Moreover, ubiquitination assays demonstrated that YL064 treatment enhanced KLF4 ubiquitination (Fig. 4J). Collectively, these findings indicate that YL064 facilitates KLF4 degradation through UPP, consistent with its inhibitory action on ATXN3 deubiquitinating activity.

### YL064 inhibits the proliferation and metastasis of breast cancer cells

**Fig. 5:**
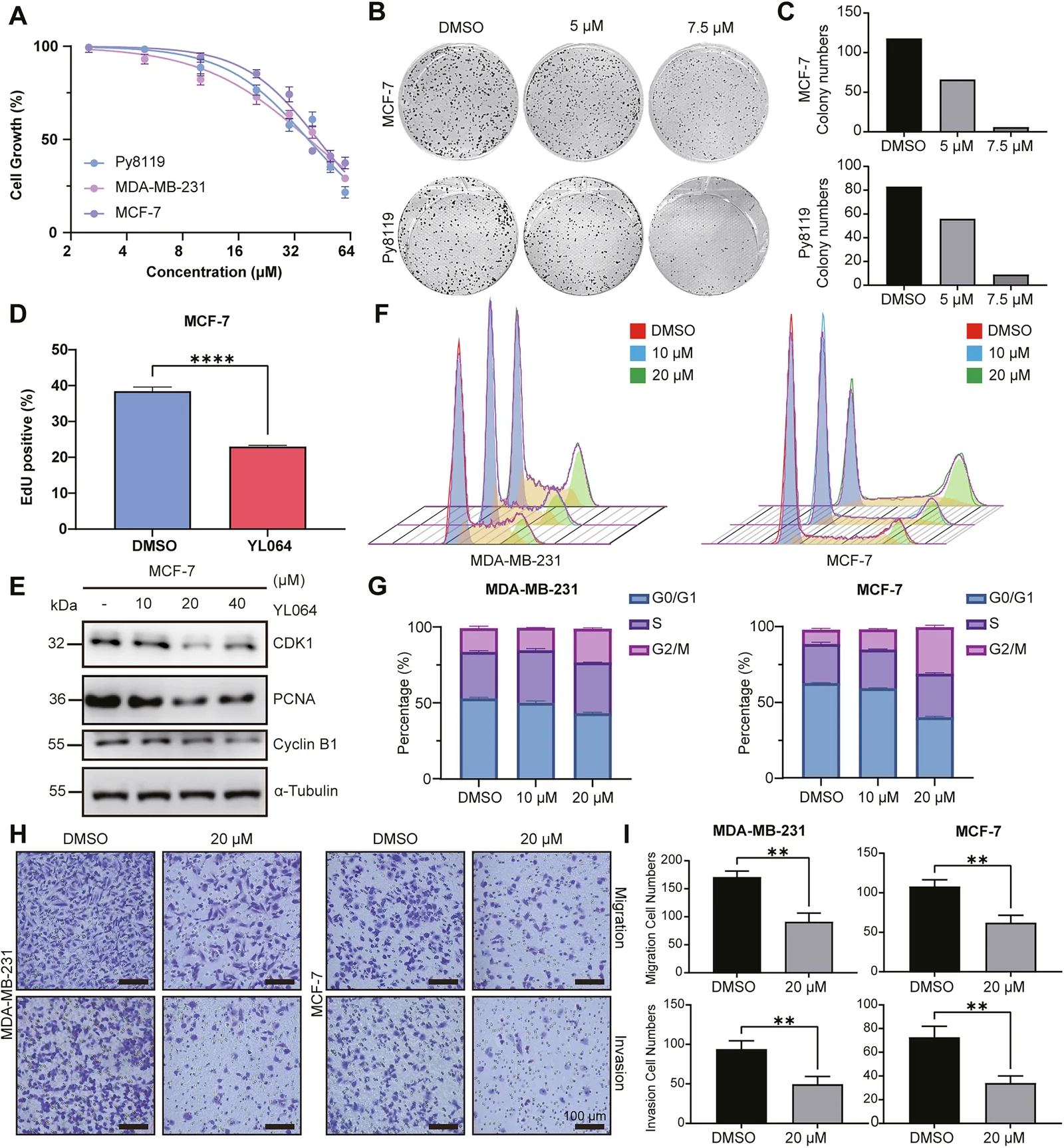
Assessment of the in vitro anticancer properties of YL064 in breast cancer. **A** Cell growth of Py8119, MDA-MB-231, and MCF-7 cells following YL064 treatment was evaluated using the CCK-8 assay (*n* = 3). Dose–response curves were plotted in GraphPad Prism 9.5, from which an average IC_50_ value of 39.71 μM was calculated (IC_50_ of Py8119 = 37.98 μM, IC_50_ of MCF-7 = 42.73 μM, IC_50_ of MDA-MB-231 = 38.42 μM). **B** Long-term proliferative capacity of Py8119 cells treated with DMSO, 5 μM YL064, or 7.5 μM YL064 for 10 days was examined using colony-formation assays. Representative colony-formation images. **C** Colonies were counted using ImageJ corresponding to panel (**B**). **D** Quantification of EdU-positive MCF-7 cells following treatment with DMSO or 15 μM YL064 (*n* = 3). **E** Immunoblot analysis of PCNA, CDK1, and Cyclin B1 expression in MCF-7 cells treated with different concentrations of YL064. **F** Flow cytometry analysis illustrating cell-cycle phase distribution in MCF-7 and Py8119 cells after exposure to YL064 (*n* = 3). Staggered offset histograms represent cells treated sequentially with DMSO, 10 μM YL064, and 20 μM YL064 from front to back. **G** Quantitative summary of cell-cycle phase proportions corresponding to the data shown in panel (**F**). **H** Representative micrographs illustrating migration and invasion of MDA-MB-231 and MCF-7 cells after exposure to DMSO or 20 μM YL064. **I** Quantitative analysis of the migration and invasion assays shown in panel (**H**). (**P < 0.01, ****P < 0.0001. Data were analyzed by Student’s *t* test).

We evaluated the cytotoxic potency of YL064 in the breast cancer cell lines Py8119, MCF-7, and MDA-MB-231. Dose–response curves were generated, yielding an average IC_50_ value of ~39.71 μM (Fig. 5A). However, the proliferation inhibitory activity of YL064 was unlikely to result from apoptosis induction. In parental and *ATXN3*-knockdown MCF-7 cells with or without YL064 treatment, there was no obvious increase in the percentage of apoptotic cells under these conditions (Supplementary Fig. S4A), and cleaved PARP1 was not detected following YL064 treatment (Supplementary Fig. S4B). To further assess its anti-proliferative effect, we conducted clonogenic assays. Compared with the control group, YL064-treated cells formed markedly fewer and smaller colonies (Fig. 5B, C). EdU incorporation analysis showed that YL064 treatment significantly reduced the percentage of EdU-positive cells, indicating impaired DNA synthesis and suppressed cell proliferation (Fig. 5D). We further examined the expression of proliferation-related markers and found that PCNA levels decreased in a dose-dependent manner following YL064 treatment (Fig. 5E). In addition, the cell-cycle regulators CDK1 and Cyclin B1 were markedly downregulated after drug treatment (Fig. 5E). Cell-cycle analysis additionally revealed that YL064 induced an accumulation of cells in the G2/M phase (Fig. 5F, G), indicating a blockade of cell-cycle progression. Collectively, these results demonstrate that YL064 effectively limits breast cancer cell proliferation.

To determine whether YL064 also affects metastatic behaviors, breast cancer cells were exposed to 20 μM YL064 for 24 h and subsequently subjected to migration and invasion assays. The number of cells traversing the transwell membrane was substantially reduced following treatment (Fig. 5H, I), suggesting that YL064 suppresses both migratory and invasive capabilities. These data indicate that YL064 exerts potent inhibitory effects on breast cancer cell growth, migration, and invasion in vitro.

### YL064 exerts anticancer effects in prostate cancer

**Fig. 6:**
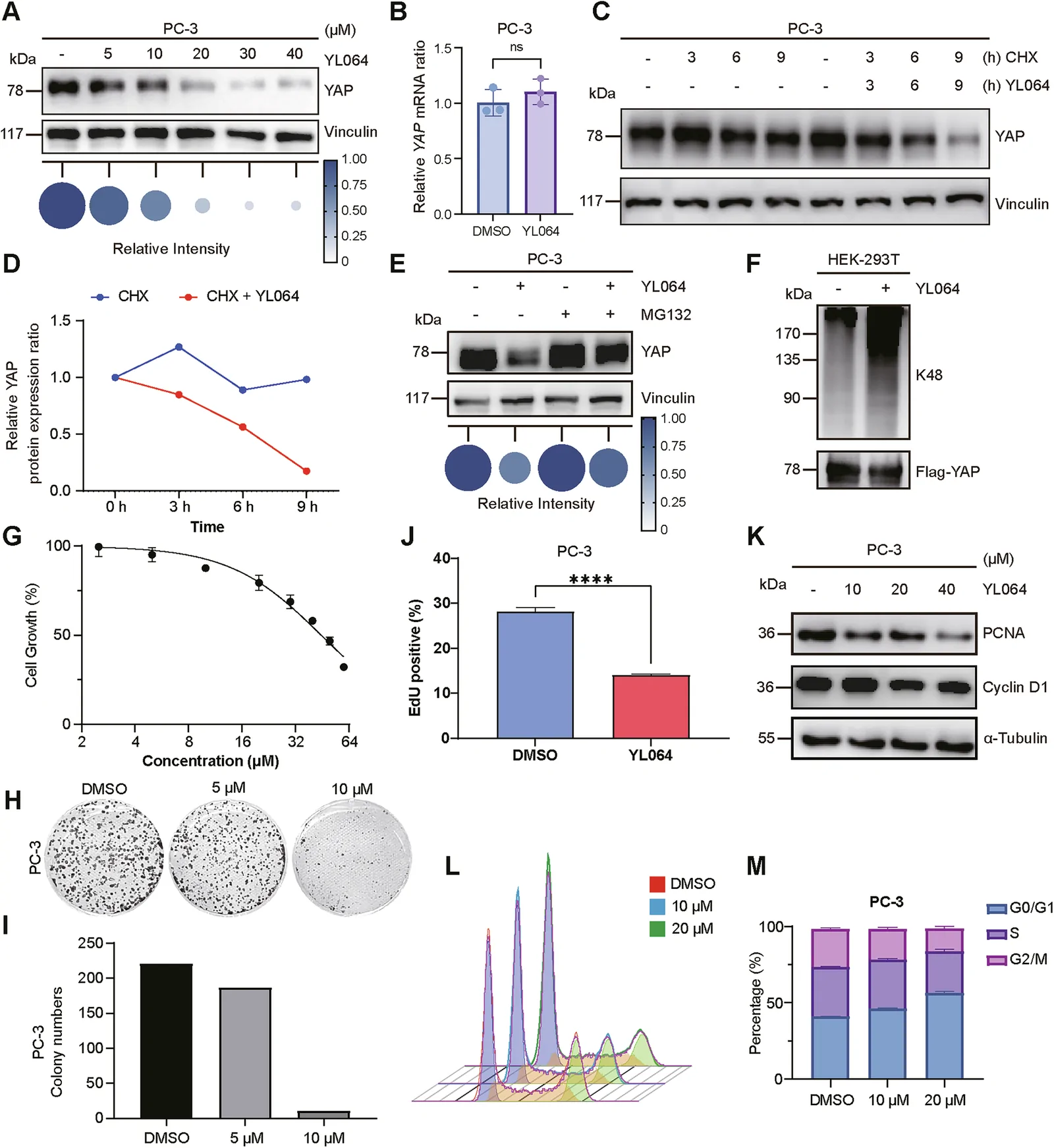
YL064 promotes YAP degradation via UPP, thereby suppressing prostate cancer progression. **A** Western blot analysis and quantification of YAP protein levels in PC-3 cells following 12 h treatment with DMSO or increasing concentrations of YL064. **B** Relative transcriptional expression of *YAP* in PC-3 cells exposed to DMSO or 20 μM YL064 for 12 h. **C** Assessment of YAP protein half-life by Western blotting. **D** Quantification of the relative YAP expression ratios obtained from (**C**). **E** Evaluation of YAP protein abundance in PC-3 cells subjected to the UPP degradation assay using Western blotting and the relative intensity is shown. **F** Detection of K48-linked ubiquitination of YAP in HEK-293T cells transfected with Flag-YAP, then treated with 5 μM MG132 for 4 h in the presence or absence of 20 μM YL064 for 12 h. **G** Dose–response curve of YL064 in PC-3 cells generated using GraphPad Prism 9.5, yielding an IC_50_ value of 44.71 μM (*n* = 3). **H** Representative images of colony formation in PC-3 cells following YL064 treatment. **I** Quantification of colony numbers in panel (**H**). **J** Quantification of EdU-positive PC-3 cells following treatment with DMSO or 15 μM YL064 (*n* = 3). **K** Immunoblot analysis of PCNA and Cyclin D1 expression in PC-3 cells treated with different concentrations of YL064. **L** Flow cytometry analysis illustrating cell-cycle phase distribution in PC-3 cells after exposure to YL064 (*n* = 3). Staggered offset histograms represent cells treated sequentially with DMSO, 10 μM YL064, and 20 μM YL064 from front to back. **M** Quantitative summary of cell-cycle phase proportions corresponding to the data shown in panel (**L**). (****P < 0.0001. Data were analyzed by Student’s *t* test).

To extend our investigation of YL064 beyond breast cancer and to determine whether its inhibitory activity toward ATXN3 is conserved in other malignancies, we next examined its effects in prostate cancer. Previous studies have demonstrated that ATXN3 facilitates prostate tumor progression by deubiquitinating and stabilizing YAP. In alignment with our observations in breast cancer models, treatment of PC-3 cells with increasing concentrations of YL064 resulted in a dose-dependent reduction of YAP protein expression (Fig. 6A). This reduction was attributable to enhanced degradation via the UPP, as confirmed by multiple biochemical assays (Fig. 6B–F).

Dose–response evaluation of YL064 in PC-3 cells yielded an IC_50_ value of 44.71 μM (Fig. 6G). Similar to the observations in breast cancer cells, the growth-inhibitory effect of YL064 in PC-3 cells was not associated with apoptosis induction, as no cleaved PARP1 was detected following treatment (Supplementary Fig. S4B). Colony formation assays showed a marked decrease in clonogenic survival following YL064 exposure, indicating strong suppression of PC-3 cell proliferative capacity (Fig. 6H, I). Moreover, in PC-3 cells, YL064 treatment similarly reduced the percentage of EdU-positive cells (Fig. 6J) and downregulated PCNA and Cyclin D1 expression (Fig. 6K). Flow cytometry analysis further demonstrated that YL064-induced G0/G1 cell-cycle arrest (Fig. 6L, M), consistent with the decreased Cyclin D1 expression and previous reports on the role of ATXN3 in cell-cycle regulation [14]. Collectively, these findings indicate that YL064 also inhibits ATXN3 activity in prostate cancer cells and impedes tumor cell proliferation by promoting YAP degradation.

### The cellular effect of YL064 is mediated by its inhibitory effect on ATXN3 activity

**Fig. 7:**
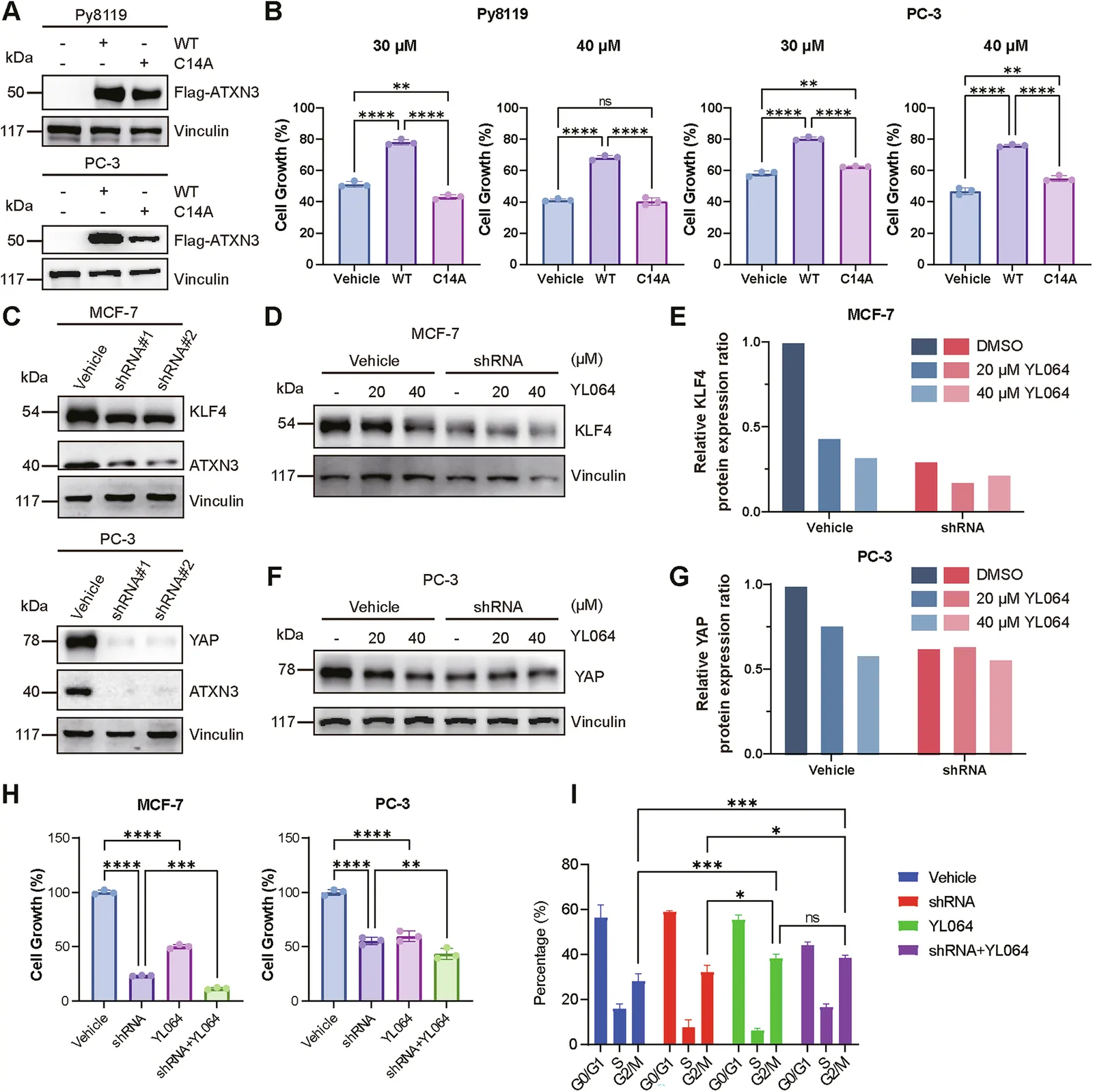
The effect of YL064 partially depends on ATXN3. **A** Western blot analysis of Flag-tagged constructs demonstrating the overexpression efficiency of ATXN3 WT and ATXN3 C14A. **B** Cell growth of parental, ATXN3 WT-overexpressing, and ATXN3 C14A-overexpressing Py8119 and PC-3 cells following YL064 treatment, assessed by the CCK-8 assay (*n* = 3). Cells treated with DMSO were used as controls and normalized to 100%. **C** Western blot analysis of ATXN3 and its corresponding substrates (KLF4 in the breast cancer cell line MCF-7 and YAP in the prostate cancer cell line PC-3) after *ATXN3* knockdown by shRNA. **D**, **F** Immunoblot analysis of KLF4 and YAP expression in parental and *ATXN3*-knockdown MCF-7 and PC-3 cells following YL064 treatment. **E**, **G** Quantification of the relative protein levels shown in (**D**) and (**F**). **H** Cell growth of parental and *ATXN3*-knockdown MCF-7 and PC-3 cells following YL064 treatment, assessed by the CCK-8 assay (*n* = 3). **I** Cell-cycle distribution of parental and *ATXN3*-knockdown MCF-7 and PC-3 cells after YL064 treatment (*n* = 3). (*P < 0.05, **P < 0.01, ***P < 0.001, ****P < 0.0001. Panel (**B**) was analyzed by one-way ANOVA, whereas the other panels were analyzed by two-way ANOVA).

The findings described above suggested that YL064 facilitated the degradation of ATXN3 substrates through UPP in both breast and prostate cancer cells, thereby exerting its anti-tumor activity. Notably, this effect was not attributed to a decrease in ATXN3 protein levels. To further confirm whether the enzyme activity of ATXN3 is critical for the anti-tumor effect of YL064, a rescue experiment was conducted. After overexpressing either the catalytically active ATXN3 WT or the catalytically inactive mutant ATXN3 C14A in Py8119, MCF-7, and PC-3 cells, the overexpression efficiency was detected (Fig. 7A). Then, the cells were treated with YL064 to test cell viability with CCK-8. The results demonstrated that overexpression of ATXN3 WT, but not ATXN3 C14A, significantly restored the cell viability suppressed by YL064 treatment (Fig. 7B). These results suggest that inhibition of the enzyme activity of ATXN3 is essential for the action of YL064.

We next knocked down *ATXN3* in MCF-7 and PC-3 cells (Fig. 7C) to further determine whether the inhibitory effect of YL064 depends on ATXN3. YL064 was then applied to parental and *ATXN3*-knockdown cells, followed by examination of ATXN3 substrates. Upon YL064 treatment, KLF4 and YAP expression were reduced only in parental cells, but not in *ATXN3*-knockdown cells (Fig. 7D–G), suggesting that the regulation of these substrates by YL064 is ATXN3-dependent. However, CCK-8 analysis showed that YL064 still suppressed cell growth in ATXN3-depleted cells (Fig. 7H). In parallel, cell-cycle arrest was further enhanced by YL064 treatment. Both *ATXN3* knockdown and YL064 treatment individually induced G2/M arrest, whereas YL064-treated *ATXN3*-knockdown MCF-7 cells exhibited a marked decrease in the G0/G1 population, accompanied by S-phase accumulation and enhanced G2/M arrest (Fig. 7I). These findings suggest that combined ATXN3 depletion and YL064 treatment disrupt cell-cycle progression. Overall, these results demonstrated that ATXN3 is a direct target of YL064 and that the anticancer effect of YL064 is partially dependent on ATXN3.

### YL064 suppressed the growth and metastasis of breast cancer cells in vivo

**Fig. 8:**
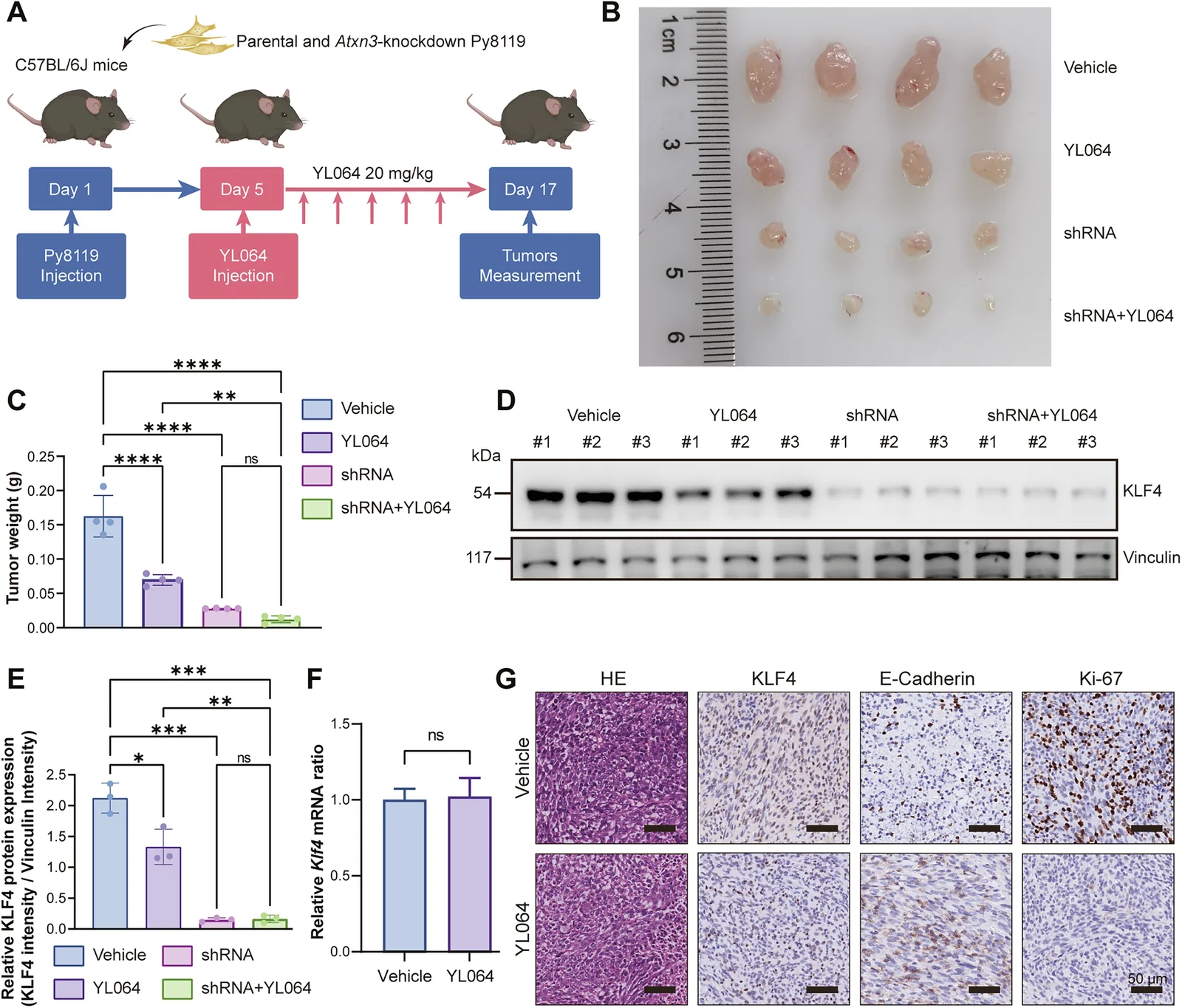
Evaluation of the therapeutic efficacy of YL064 in vivo. **A** Schematic illustration of the in vivo experimental design. Parental and *Atxn3*-knockdown Py8119 cells (5 × 10^5^) were orthotopically implanted into the mammary fat pads of C57BL/6J mice. Once tumors were established, animals bearing TNBC tumors received either vehicle or YL064 (20 mg/kg) treatment every other day for a total of 6 administrations (*n* = 4). **B** Representative images of excised tumors. **C** Corresponding quantification of tumor weights (*n* = 4). **D** Western blot analysis of KLF4 protein expression in tumor tissues (*n* = 3). **E** Quantification of relative KLF4 protein expression in panel (**D**), calculated as the ratio of KLF4 intensity to Vinculin intensity (*n* = 3). **F** Transcriptional levels of *Klf4* were determined after homogenization of tumor samples. **G** Representative histological and immunohistochemical images of tumor sections from vehicle and YL064-treated groups, stained with H&E, KLF4, E-cadherin, and Ki-67. Scale bar = 50 μm. (*P < 0.05, **P < 0.01, ***P < 0.001, ****P < 0.0001. Panel (**F**) was analyzed by Student’s *t* test, whereas the other panels were analyzed by two-way ANOVA).

Based on the in vitro data, we next assessed the in vivo effect of YL064 using a murine model of breast cancer. Parental and *Atxn3*-knockdown Py8119 breast cancer cells were orthotopically implanted into the mammary fat pads of C57BL/6J mice. Tumor nodules became palpable ~5 days post-inoculation, at which point the animals were randomly assigned to either a control group or a YL064-treated group. Mice in the treatment cohort received intraperitoneal injections of YL064 on alternate days, and the experiment was concluded 17 days after tumor implantation (Fig. 8A).

Comparative analysis of tumor size (Fig. 8B) and mass (Fig. 8C) revealed that YL064 administration suppressed tumor growth in treated mice. Consistent with the in vitro findings, YL064 retained its tumor-suppressive effect even after *Atxn3* knockdown. Protein and mRNA analyses from excised tumors showed that KLF4 protein levels were substantially reduced in the YL064-treated group (Fig. 8D, E), while *Klf4* mRNA expression remained unchanged (Fig. 8F), indicating the post-translational regulation involved. Meanwhile, in *Atxn3*-knockdown tumors, YL064 was unable to further down-regulate KLF4, suggesting that ATXN3 is the target of YL064 in vivo (Fig. 8D, E). Immunohistochemical staining further confirmed these observations, showing increased E-cadherin expression together with diminished levels of KLF4 and the proliferation marker Ki-67 in response to YL064 administration (Fig. 8G). These alterations indicated that YL064 effectively restrained tumor progression and metastatic potential.

## Discussion

Despite the well-established oncogenic role of ATXN3 in multiple cancers, effective inhibitors targeting this DUB remain undeveloped. Our study addresses this critical gap by identifying YL064, a novel sinomenine derivative, as a potent and selective ATXN3 inhibitor. Through comprehensive biochemical and cellular analyses, we demonstrate that YL064 directly binds ATXN3, inhibits its DUB activity, and promotes proteasomal degradation of key oncogenic substrates, including KLF4 [12] in breast cancer and YAP in prostate cancer [14]. These molecular effects translate to significant suppression of tumor cell proliferation, migration, and invasion.

Building on our previous work characterizing YL064 as a multi-target inhibitor of STAT3 [20] and c-MYC [21], we now establish ATXN3 as another important molecular target through multiple lines of evidence: [1] direct inhibition of ATXN3 catalytic activity in biochemical assays; [2] confirmation of physical interaction using thermal shift assays (TSA/CETSA); and [3] functional validation through ATXN3 substrate degradation. Importantly, the anti-tumor effects of YL064 were partially rescued by ATXN3 overexpression, establishing a clear mechanism of action. Meanwhile, knockdown of *ATXN3* abolished the regulatory effects of YL064 on KLF4 and YAP protein stability, confirming that these downstream effects are largely ATXN3-dependent.

Structural optimization of YL064, particularly through B-ring modification, confers significant pharmacological advantages over its parent compound sinomenine. Molecular docking reveals enhanced binding interactions including a critical hydrogen bond with H124 and π-π stacking with F10 in ATXN3’s Josephin domain, along with improved hydrophobic contacts. We generated mutant ATXN3 proteins and carried out the TSA assay to see if the interaction was hindered (Supplementary Fig. S2A, B). It should be noted that TSA primarily reflects changes in protein thermal stability and provides indirect evidence of protein-ligand interactions. Therefore, the current results are insufficient to definitively conclude that F10 and H124 are the direct binding sites between ATXN3 and YL064. Moreover, the mutations themselves significantly altered the intrinsic stability (Supplementary Fig. S2C) and deubiquitinating activity (Fig. 2G) of ATXN3, which may also influence the TSA outcomes. Nevertheless, the observed effects of these mutations on protein stability and enzymatic activity (Fig. 2G) suggest that F10 and H124 may play non-negligible roles in maintaining ATXN3 function and mediating its interaction with YL064. Further structural and biochemical studies are required to elucidate the precise mechanisms and binding mode involved.

Our findings have particular relevance for breast and prostate cancers—the most prevalent malignancies in women and men, respectively. In breast cancer models, YL064 specifically promoted KLF4 degradation without affecting mRNA levels, while similar effects were observed for YAP in prostate cancer. Given ATXN3’s documented roles in additional malignancies, including thyroid [22] and hepatocellular carcinomas [23], YL064 may have broader therapeutic applications against ATXN3-dependent cancers. While our data clearly establish ATXN3 inhibition as a major mechanism underlying YL064 activity, YL064 still retained partial anti-proliferative effects in ATXN3-depleted cells both in vitro and in vivo, suggesting that its multi-target properties, including previously reported effects on STAT3 [20] and c-MYC [21], may also contribute to its overall anti-tumor efficacy.

In conclusion, our study provides the first pharmacological evidence that targeted inhibition of ATXN3 can effectively disrupt oncoprotein stability and suppress tumor progression. YL064 represents a structurally and mechanistically novel lead compound in the growing field of DUB inhibitors. Future development should focus on optimizing YL064’s selectivity and pharmacokinetic properties while exploring its potential in combination therapies and additional ATXN3-driven malignancies. These findings open new avenues for targeting protein degradation in cancer therapy.

## Materials and methods

### Protein purification

Transformed full-length ATXN3 with pET-SUMO as a carrier and His as a tag into Escherichia coli BL21 and amplified in YT medium. Stop the amplification when the O.D. value arrived at 0.6–1.0 induced by 0.2 mM Isopropyl β-D-Thiogalactoside (IPTG), 12 h, 24 °C for protein generation. Under high pressure, bacteria were crushed, and His-ATXN3 was purified by affinity chromatography through a nickel column. His-tag was enzymatically cut from ATXN3 by SENP2, and the purified protein ATXN3 was obtained.

### In vitro enzymatic assays

The DUB activity of ATXN3 was reconstructed by using ubiquitin-7-amido-4-methylcoumarin (Ub-AMC) as substrate. With enzyme activity, ATXN3 could cleave and release dissociative Ub. Through detecting the fluorescence of Ub, the activity was indirectly presented. Similarly, the potent inhibitors were filtered in this way.

### Cell lines and cell culture

Human breast cancer cell lines MDA-MB-231 and MCF-7 were obtained from ATCC and cultured in DMEM (G4511-500ML, Servicebio, China) with 10% FBS (F101-01, Vazyme, China) and 1% Penicillin Streptomycin (MA0110, MeilunBio, China). Mouse breast cancer cell line Py8119 was obtained from ATCC and cultured in DMEM/F-12 (G4610-500ML, Servicebio, China) with 10% FBS and 1% Penicillin Streptomycin. Human prostate cancer cell line PC-3 was obtained from ATCC and cultured in DMEM/F-12 with 10% FBS and 1% Penicillin Streptomycin. HEK-293T cells were obtained from ATCC and cultured in DMEM with 10% FBS and 1% Penicillin Streptomycin. All cells were cultured at 37 °C in a humidified atmosphere of 5% CO_2_. All cell stocks were authenticated by STR profiling and routinely tested for mycoplasma contamination and showed negative.

### Western blot analysis

Purified proteins or proteins extracted from cells were split by using lysis buffer, which contains detergents and protease inhibitors, and heated in SDS loading buffer for 5–10 min at 98 °C three times. Equal protein samples were SDS-PAGE separated, then transferred to nitrocellulose membranes using an electro-blotting apparatus. Membranes were blocked in PBS-T with 5% skimmed milk, then incubated with primary antibodies overnight at 4 °C. After PBS-T washing, membranes were incubated with horseradish peroxidase-conjugated secondary antibodies for 1 h at room temperature. Protein expression was visualized using the ECL kit.

Vinculin and α-Tubulin served as internal references for normalization of protein expression in the corresponding experiments. The antibodies used in the present study were as follows: ATXN3 antibody (Proteintech, 13505-1-AP; 1:2000), α-Tubulin antibody (Proteintech, 80762-1-RR; 1:2000), YAP antibody (Cell signaling technology, 4912; 1:2000), PCNA antibody (Proteintech, 10205-2-AP; 1:2000), Vinculin antibody (ABclonal, A2752; 1:1000), KLF4 antibody (HUABIO, ET1702-71; 1:2000), His-Tag antibody (Cell Signaling Technology, 2365; 1:2000), Cyclin B1 antibody (Proteintech, 55004-1-AP; 1:1000), Cyclin D1 antibody (Proteintech, 60186-1-Ig; 1:2500), CDK1 antibody (HUABIO, ER31213; 1:500), Flag-Tag antibody (Smart-Lifescience, SLAB0102; 1:2000).

### Thermal shift assay (TSA) and cellular thermal shift assay (CETSA)

TSA and CETSA utilize the concept that target proteins would get stabilized or unstable when drug molecules bind in vitro or in cells to explore the interaction between target proteins and drug molecules [24]. For CETSA, cells were lysed in PBS, involving protease inhibitors, by ultrasound, and the cell suspension was centrifuged at 4 °C, 12,000 rpm. The supernatant was incubated with dimethylsulfoxide (DMSO) and target drug molecules, respectively, at 37 °C for 2 h to bring the target protein and drug molecules into full alignment. The mixture was heated in a thermal gradient and tested by Western Blot analysis. Unlike CETSA, TSA was performed using purified proteins rather than cell lysates, including proteins obtained from prokaryotic expression systems or eukaryotic expression systems (with the eukaryotic-purified protein designated as Flag-ATXN3).

### Biacore surface plasmon resonance (SPR) assay

The interaction between YL064 and ATXN3 was characterized using a Biacore 8 K SPR system (Cytiva). Recombinant ATXN3 protein was prepared in acetate buffer (pH 4.0) and covalently coupled to a CM5 sensor chip via an amine-coupling strategy using the manufacturer’s protocol (Cytiva, BR100050). All measurements were conducted at 25 °C using PBS-P+ as the running buffer. A blank flow cell without immobilized protein served as the reference control. Increasing concentrations of YL064 were then flowed across the chip surface to monitor real-time binding responses. After each injection cycle, the sensor surface was regenerated with 50% DMSO. Binding kinetics were analyzed using the Biacore evaluation software, and the equilibrium dissociation constant (KD) was calculated by nonlinear fitting of the response curves.

### RT-qPCR

With the help of FastPure Cell/Tissue Total RNA Isolation Kit V2 (RC112-01, Vazyme) according to the manufacturer’s instructions, total RNA was extracted from breast cancer cells and tumor tissues. Complementary DNA was transcribed through a reverse transcription kit according to the manufacturer’s instructions. *ACTB* was applied as an internal control, while the relative mRNA expression was analyzed by the 2^−ΔΔ*Ct*^ method. The relative sequences have been listed in the supplementary materials.

### CCK-8 proliferation assay

Tumor cells were incubated with different concentrations of YL064 or DMSO for 24 h. Cell growth was assayed using a Cell Counting Kit-8 according to the manufacturer’s instructions. The cell’s response to each concentration is measured, and the data are plotted to generate a dose–response curve. The IC_50_ value is then determined from this curve.

### Clonogenic cell survival assay

YL064-treated or control MCF-7, Py8119 and PC-3 cells were seeded onto 6-well cell culture dishes. The culture medium was refreshed with fresh medium every 3 days. After 10 days of treatment, the medium was aspirated, and cell colonies were fixed and stained with crystal violet (0.1% in 20% methanol). Surviving colonies were enumerated by ImageJ.

### EdU incorporation assay

Cell proliferation was assessed using an EdU Cell Proliferation Kit (CX002, Epizyme, China) according to the manufacturer’s instructions. Briefly, cells were treated with YL064 (15 μM) or DMSO control overnight, followed by incubation with EdU for 2 h at 37 °C. The percentage of EdU-positive cells was analyzed by flow cytometry, and data were processed using FlowJo software.

### Transwell assay

MCF-7 and MDA-MB-231 cells were treated with 20 μM YL064 or DMSO for 24 h in serum-free culture medium. After the treatment, cells were digested and mixed with serum-free culture medium at a density of 5 × 10^6^ cells/mL. 100 μL of cell suspension was plated in the upper insert of a Transwell chamber containing an 8 μm polycarbonate membrane, with or without Matrigel coating. In the meantime, in the lower Transwell chamber, 600 μL culture medium with serum was filled. After seeding for 24 h, the inserts were fixed and stained with crystal violet (0.1% in 20% methanol). Cells on the upper side of the membrane were removed and we counted the cells on the bottom side of the pore membrane across 3 random microscopic fields. The migrated and invaded cells were enumerated by ImageJ and analyzed by GraphPad Prism 9.5.

### Plasmids and transfection

The full-length WT and C14A mutant constructs of *ATXN3* were obtained by PCR amplification of the full-length sequence of human *ATXN3* and subcloned into pLVX.1-puro vector. Sequences of the small hairpin RNAs (shRNAs) used for *ATXN3* knockdown in this study are listed in the Supplementary Table S2. To produce lentiviruses, HEK-293T cells were co-transfected with the plasmids above and the packaging plasmids, psPAX2 and PMD2G. PEI served as the transfection reagent. Supernatants were collected 48–72 h after transfection and concentrated by ultra-high-speed centrifugation at 4 °C, 28,000 rpm. Then, Py8119 and PC-3 cells were infected by the above lentivirus in the presence of 8 μg/mL of polybrene. Stable cell clones were selected by 2.0 μg/mL of puromycin 48 h postinfection.

### Molecular docking

YL064 was prepared and optimized using LigPrep (LigPrep, Schrödinger, LLC, New York, NY, 2021) with OPLS4 force field [25]. Appropriate protonation and ionization states of YL064 were assigned at pH 7.4 ± 1.0 using Epik [26].

The three-dimensional structure of ATXN3 (PDB ID: 2AGA, https://www.rcsb.org/) was used for docking. It was submitted to protein preparation wizard [26] of Schrödinger Suite for hydrogen atom addition, bond order assignment, protonation state generation, hydrogen bond sampling at pH 7.4 using PROPKA [27, 28], and energy minimization with OPLS4 force field [25]. Docking grids were generated using the centroid of Josephin binding site [29] (residues including H11, Q14, E15, L18, C19, A20, H22, N25, N26, F33, H124, W125, L141, and T143) of ATXN3 with a bounding box size of 14 Å in X, Y, and Z axes. Both Standard Precision (SP) and Extra Precision (XP) docking modes were employed to thoroughly explore the binding conformations. And the most favorable pose was identified based on their positions and interactions from all the outputs. UCSF ChimeraX [30] was utilized for structure visualization and figure preparation.

### In vivo experiments

C57BL/6J mice, around 6–8 weeks old, were adopted as the subjects of in vivo experiments. All mice dwelt in individually ventilated cages with 12-h light and dark cycles at 20–24 °C and 30–70% relative humidity, had free access to water and food. Py8119 cells with or without *Atxn3* knockdown (5 × 10^5^ cells per mouse) were injected into mice. 17 days after tumor implantation, tumors were harvested for further analysis. For drug efficacy studies, five days after tumor implantation, mice were grouped randomly. YL064 dissolved in a vehicle containing 5% DMSO, 30% PEG300, 10% Tween 80, and 55% ddH_2_O was administered via intraperitoneal injection. Mice received a total of 6 treatments.

### Statistical analysis

All the experiments were repeated at least 3 times. The statistical methods used for each experiment are specified in the corresponding figure legends. All images of Wound-healing assay, clonogenic survival assay, and western blot were analyzed with the help of ImageJ, and the data were presented by GraphPad Prism 9.5. *P < 0.05, **P < 0.01, ***P < 0.001, ****P < 0.0001.

## Supplementary information

SUPPLEMENTAL MATERIAL

Original Data

## Data Availability

The data supporting the findings of this study are available within the article and its Supplementary files.

## References

[1] Lei H, Wang J, Hu J, Zhu Q, Wu Y. Deubiquitinases in hematological malignancies. Biomark Res. 2021;9:66. 10.1186/s40364-021-00320-w PMC8401176 34454635

[2] Burnett B, Li F, Pittman RN. The polyglutamine neurodegenerative protein ataxin-3 binds polyubiquitylated proteins and has ubiquitin protease activity. Hum Mol Genet. 2003;12:3195–205. 10.1093/hmg/ddg344 14559776

[3] Paulson H. Machado-Joseph disease/spinocerebellar ataxia type 3. Handb Clin Neurol. 2012;103:437–49. 10.1016/B978-0-444-51892-7.00027-9 PMC3568768 21827905

[4] Singh AN, Oehler J, Torrecilla I, Kilgas S, Li S, Vaz B, et al. The p97-Ataxin 3 complex regulates homeostasis of the DNA damage response E3 ubiquitin ligase RNF8. EMBO J. 2019;38:e102361. 10.15252/embj.2019102361 PMC6826192 31613024

[5] Tu Y, Liu H, Zhu X, Shen H, Ma X, Wang F, et al. Ataxin-3 promotes genome integrity by stabilizing Chk1. Nucleic Acids Res. 2017;45:4532–49. 10.1093/nar/gkx095 PMC5416811 28180282

[6] Evert BO, Araujo J, Vieira-Saecker AM, de Vos RA, Harendza S, Klockgether T, et al. Ataxin-3 represses transcription via chromatin binding, interaction with histone deacetylase 3, and histone deacetylation. J Neurosci Off J Soc Neurosci. 2006;26:11474–86. 10.1523/JNEUROSCI.2053-06.2006 PMC6674535 17079677

[7] Hernández-Carralero E, Cabrera E, Rodríguez-Torres G, Hernández-Reyes Y, Singh AN, Santa-María C, et al. ATXN3 controls DNA replication and transcription by regulating chromatin structure. Nucleic Acids Res. 2023;51:5396–413. 10.1093/nar/gkad212 PMC10287915 36971114

[8] Rodrigues AJ, do Carmo Costa M, Silva TL, Ferreira D, Bajanca F, Logarinho E, et al. Absence of ataxin-3 leads to cytoskeletal disorganization and increased cell death. Biochim Biophys Acta. 2010;1803:1154–63. 10.1016/j.bbamcr.2010.07.004 20637808

[9] Liu H, Li X, Ning G, Zhu S, Ma X, Liu X, et al. The Machado-Joseph disease deubiquitinase ataxin-3 regulates the stability and apoptotic function of p53. PLoS Biol. 2016;14:e2000733. 10.1371/journal.pbio.2000733 PMC5112960 27851749

[10] Pfeiffer A, Luijsterburg MS, Acs K, Wiegant WW, Helfricht A, Herzog LK, et al. Ataxin-3 consolidates the MDC1-dependent DNA double-strand break response by counteracting the SUMO-targeted ubiquitin ligase RNF4. EMBO J. 2017;36:1066–83. 10.15252/embj.201695151 PMC5391139 28275011

[11] Li D, Zhang T, Lai J, Zhang J, Wang T, Ling Y, et al. MicroRNA‑25/ATXN3 interaction regulates human colon cancer cell growth and migration. Mol Med Rep. 2019;19:4213–21. 10.3892/mmr.2019.10090 PMC6471560 30942397

[12] Zou H, Chen H, Zhou Z, Wan Y, Liu Z. ATXN3 promotes breast cancer metastasis by deubiquitinating KLF4. Cancer Lett. 2019;467:19–28. 10.1016/j.canlet.2019.09.012 31563563

[13] Wu X, Zhang X, Liu P, Wang Y. Involvement of Ataxin-3 (ATXN3) in the malignant progression of pancreatic cancer via deubiquitinating HDAC6. Pancreatology. 2023;23:630–41. 10.1016/j.pan.2023.06.011 37460341

[14] Wu L, Ou Z, Liu P, Zhao C, Tong S, Wang R, et al. ATXN3 promotes prostate cancer progression by stabilizing YAP. Cell Commun Signal. 2023;21:152. 10.1186/s12964-023-01073-9 PMC10286397 37349820

[15] Jiang ZM, Zeng SL, Huang TQ, Lin Y, Wang FF, Gao XJ, et al. Sinomenine ameliorates rheumatoid arthritis by modulating tryptophan metabolism and activating aryl hydrocarbon receptor via gut microbiota regulation. Sci Bull. 2023;68:1540–55. 10.1016/j.scib.2023.06.027 37422372

[16] Zheng X, Li W, Xu H, Liu J, Ren L, Yang Y, et al. Sinomenine ester derivative inhibits glioblastoma by inducing mitochondria-dependent apoptosis and autophagy by PI3K/AKT/mTOR and AMPK/mTOR pathway. Acta Pharm Sin B. 2021;11:3465–80. 10.1016/j.apsb.2021.05.027 PMC8642618 34900530

[17] Zhu L, Chen C, Cai Y, Li Y, Gong L, Zhu T, et al. Identification of a ferritinophagy inducer via sinomenine modification for the treatment of colorectal cancer. Eur J Med Chem. 2024;268:116250. 10.1016/j.ejmech.2024.116250 38417218

[18] Li D, Zhong Z, Ko CN, Tian T, Yang C. From mundane to classic: sinomenine as a multi-therapeutic agent. Br J Pharmacol. 2025;182:2159–80. 10.1111/bph.16267 37846470

[19] Wei CJ, Xu F, Shi MJ, Hu JW, Wang JJ, Zhen B, et al. Synthesis and anti-tumor activities of sinomenine derivatives on rings A and C. J Asian Nat Prod Res. 2018;20:277–91. 10.1080/10286020.2017.1386659 29090602

[20] Wang Y, Wu L, Cai H, Lei H, Ma CM, Yang L, et al. YL064 directly inhibits STAT3 activity to induce apoptosis of multiple myeloma cells. Cell Death Discov. 2018;4:44. 10.1038/s41420-018-0108-8 PMC6170385 30302278

[21] Shan H, Cao Y, Xiao X, Liu M, Wu Y, Zhu Q, et al. YL064 activates proteasomal-dependent degradation of c-Myc and synergistically enhances the anti-tumor activity of ABT-199 in diffuse large B cell lymphoma. Signal Transduct Target Ther. 2020;5:116. 10.1038/s41392-020-00236-1 PMC7338474 32632092

[22] Zhuang S, Xie J, Zhen J, Guo L, Hong Z, Li F, et al. The deubiquitinating enzyme ATXN3 promotes the progression of anaplastic thyroid carcinoma by stabilizing EIF5A2. Mol Cell Endocrinol. 2021;537:111440. 10.1016/j.mce.2021.111440 34428509

[23] Peng Y, Nie H, Kang K, Li X, Tao Y, Zhou Y. The deubiquitinating enzyme ATXN3 promotes hepatocellular carcinoma progression by stabilizing TAZ. Cancer Gene Ther. 2025;32:136–45. 10.1038/s41417-024-00869-2 39672915

[24] Almqvist H, Axelsson H, Jafari R, Dan C, Mateus A, Haraldsson M, et al. CETSA screening identifies known and novel thymidylate synthase inhibitors and slow intracellular activation of 5-fluorouracil. Nat Commun. 2016;7:11040. 10.1038/ncomms11040 PMC4820820 27010513

[25] Lu C, Wu C, Ghoreishi D, Chen W, Wang L, Damm W, et al. OPLS4: improving force field accuracy on challenging regimes of chemical space. J Chem Theory Comput. 2021;17:4291–300. 10.1021/acs.jctc.1c00302 34096718

[26] Shelley JC, Cholleti A, Frye LL, Greenwood JR, Timlin MR, Uchimaya M. Epik: a software program for pKa prediction and protonation state generation for drug-like molecules. J Comput Aided Mol Des. 2007;21:681–91. 10.1007/s10822-007-9133-z 17899391

[27] Olsson MHM, Søndergaard CR, Rostkowski M, Jensen JH. PROPKA3: consistent treatment of internal and surface residues in empirical pKa predictions. J Chem Theory Comput. 2011;7:525–37. 10.1021/ct100578z 26596171

[28] Søndergaard CR, Olsson MHM, Rostkowski M, Jensen JH. Improved treatment of ligands and coupling effects in empirical calculation and rationalization of pKa values. J Chem Theory Comput. 2011;7:2284–95. 10.1021/ct200133y 26606496

[29] Nicastro G, Menon RP, Masino L, Knowles PP, McDonald NQ, Pastore A. The solution structure of the Josephin domain of ataxin-3: structural determinants for molecular recognition. Proc Natl Acad Sci USA. 2005;102:10493–8. 10.1073/pnas.0501732102 PMC1180765 16020535

[30] Pettersen EF, Goddard TD, Huang CC, Meng EC, Couch GS, Croll TI, et al. UCSF ChimeraX: structure visualization for researchers, educators, and developers. Protein Sci. 2021;30:70–82. 10.1002/pro.3943 PMC7737788 32881101

