# Supplementary material for: Identification of YL064 as a novel inhibitor of ATXN3 to suppress tumor growth and metastasis by promoting degradation of oncogenic substrates
Source: Cell Death Dis. 2026 Aug 6;17(1):934. doi: 10.1038/s41419-026-09171-5 (PMC13728240; doi:10.1038/s41419-026-09171-5)

Fig. 2

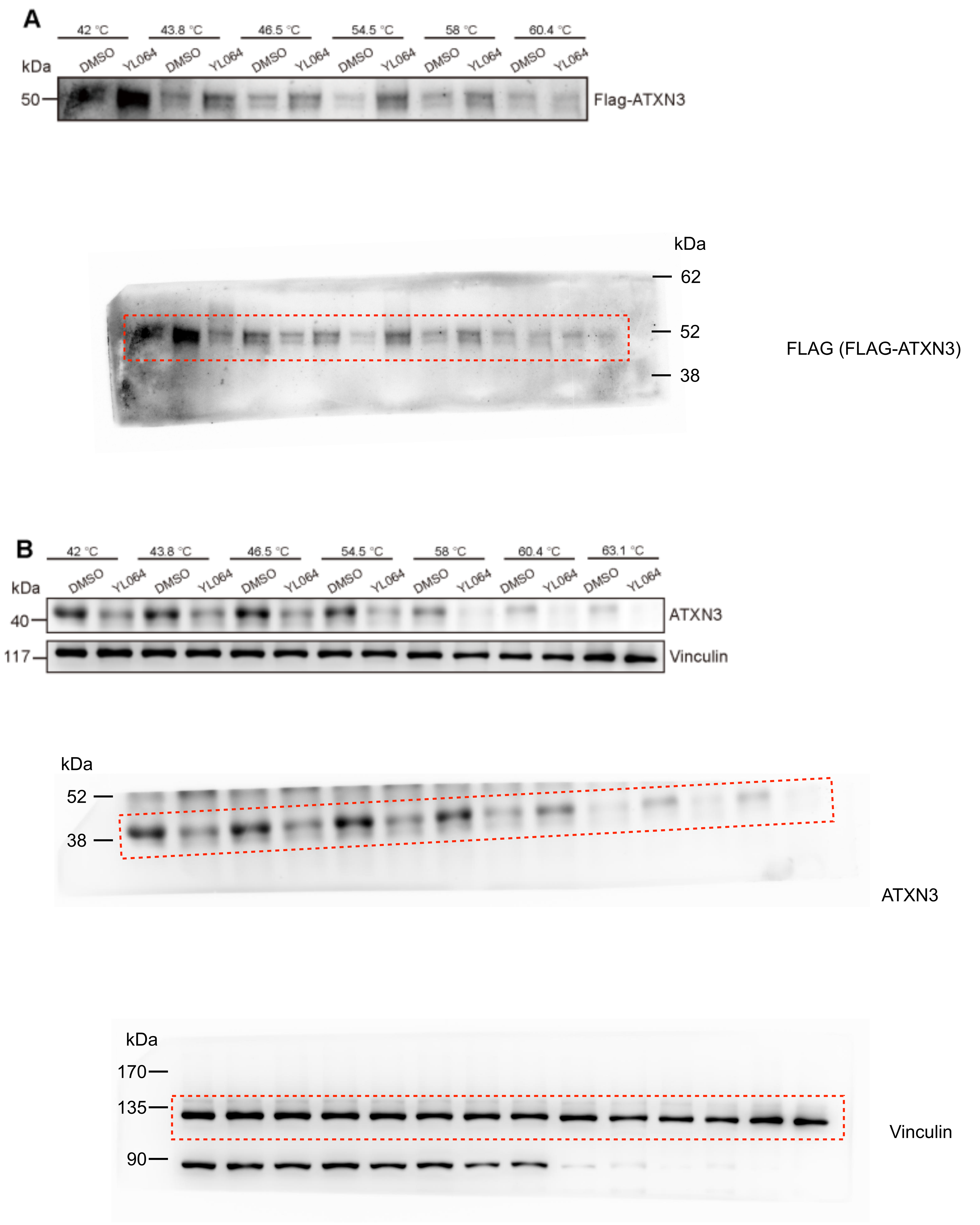
A
42 °C
43.8 °C
46.5 °C
54.5 °C
58 °C
60.4 °C
DMSO
YL064
kDa
50
Flag-ATXN3
kDa
62
52
38
FLAG (FLAG-ATXN3)
B
42 °C
43.8 °C
46.5 °C
54.5 °C
58 °C
60.4 °C
63.1 °C
DMSO
YL064
kDa
40
117
ATXN3
Vinculin
kDa
52
38
ATXN3
kDa
170
135
90
Vinculin

Fig. 3

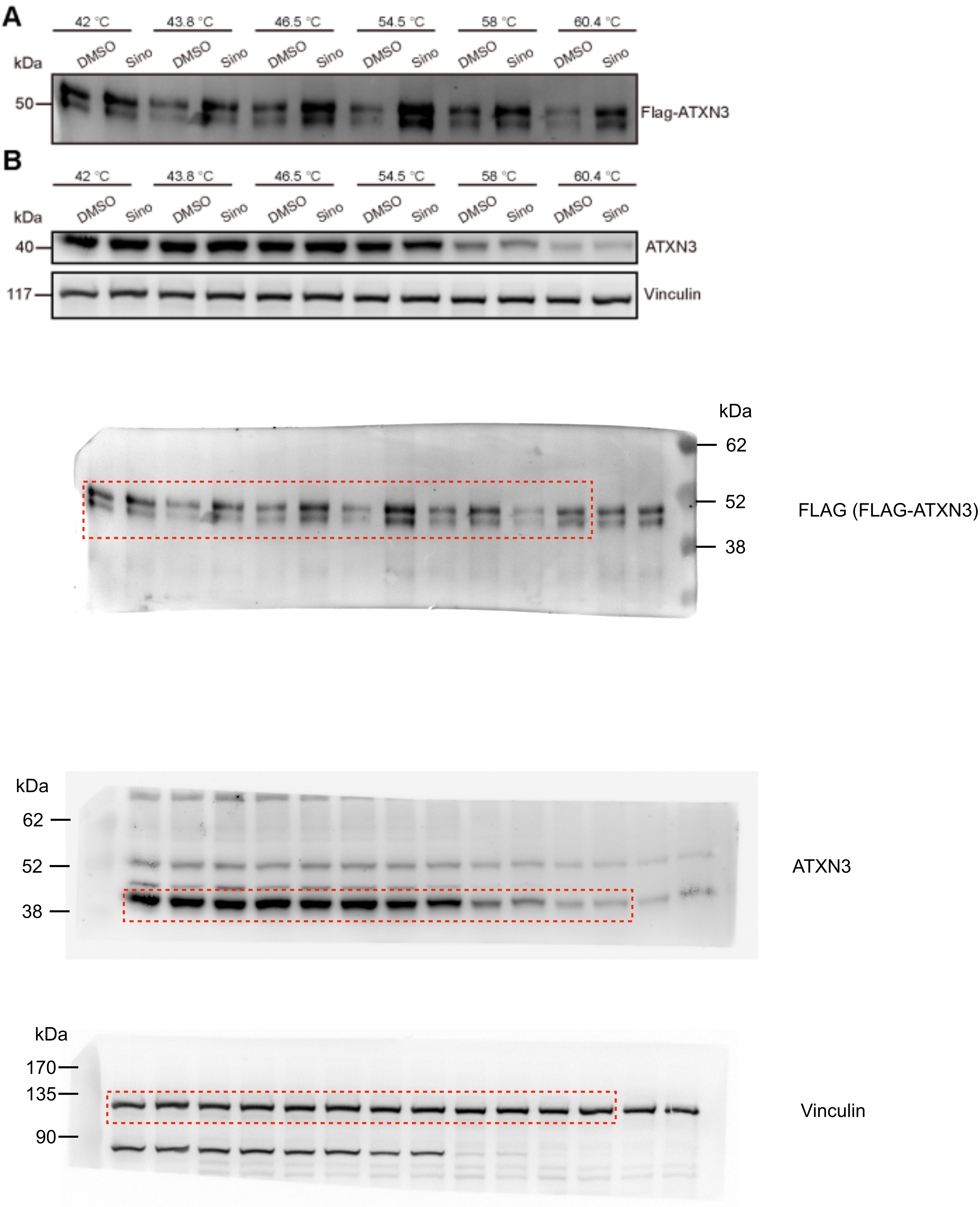

A
42 °C
43.8 °C
46.5 °C
54.5 °C
58 °C
60.4 °C
kDa
DMSO
Sino
50
Flag-ATXN3
B
42 °C
43.8 °C
46.5 °C
54.5 °C
58 °C
60.4 °C
kDa
DMSO
Sino
40
ATXN3
117
Vinculin
kDa
62
52
38
FLAG (FLAG-ATXN3)
kDa
62
52
38
ATXN3
kDa
170
135
90
Vinculin

Fig. 4

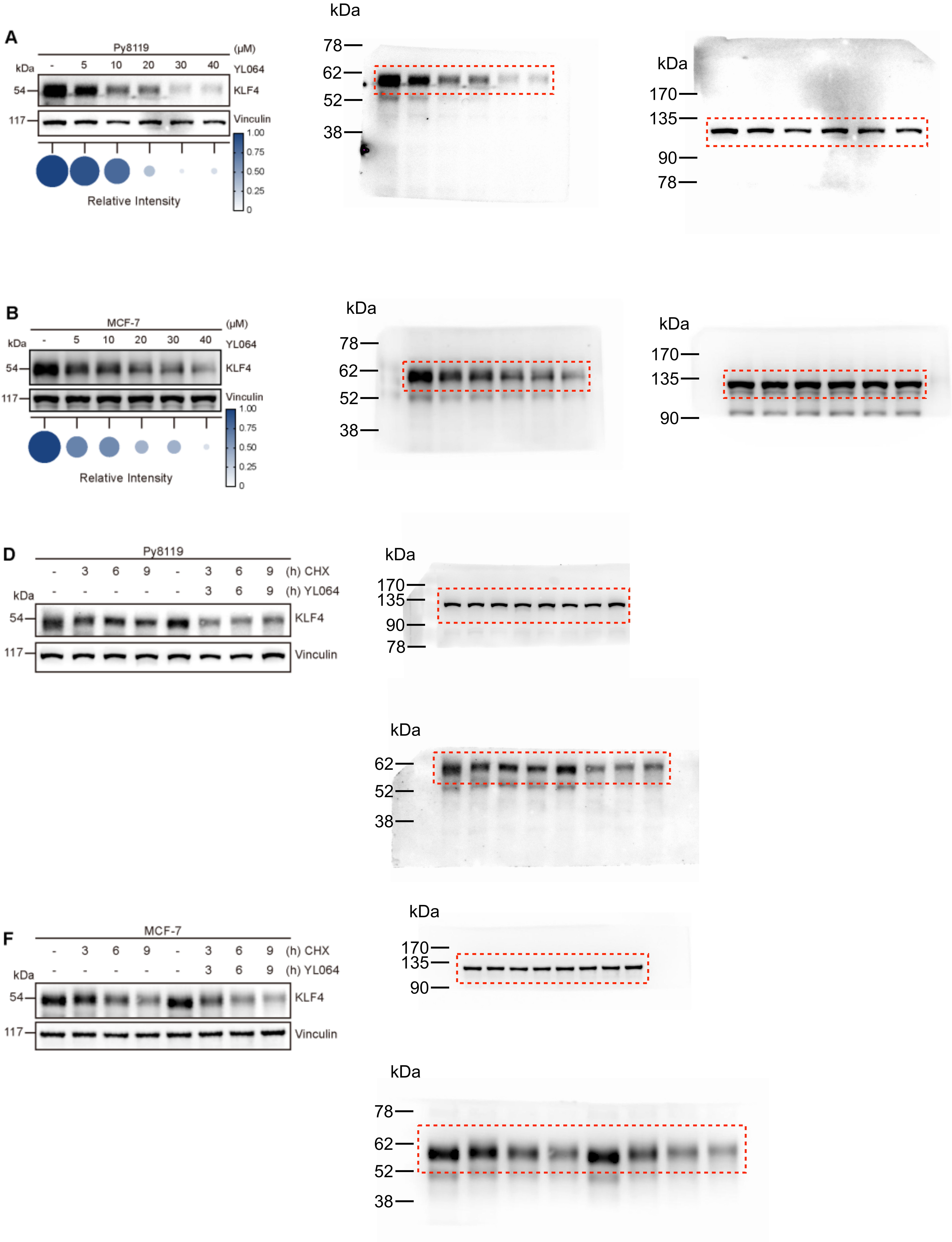
A
Py8119
(μM)
kDa
- 5 10 20 30 40
YL064
54
KLF4
117
Vinculin
1.00
0.75
0.50
0.25
0
Relative Intensity
kDa
78
62
52
38
kDa
170
135
90
78
B
MCF-7
(μM)
kDa
- 5 10 20 30 40
YL064
54
KLF4
117
Vinculin
1.00
0.75
0.50
0.25
0
Relative Intensity
kDa
78
62
52
38
kDa
170
135
90
D
Py8119
- 3 6 9 - 3 6 9 (h) CHX
- - - - - 3 6 9 (h) YL064
kDa
54
KLF4
117
Vinculin
kDa
170
135
90
78
kDa
62
52
38
F
MCF-7
- 3 6 9 - 3 6 9 (h) CHX
- - - - - 3 6 9 (h) YL064
kDa
54
KLF4
117
Vinculin
kDa
170
135
90
kDa
78
62
52
38

H

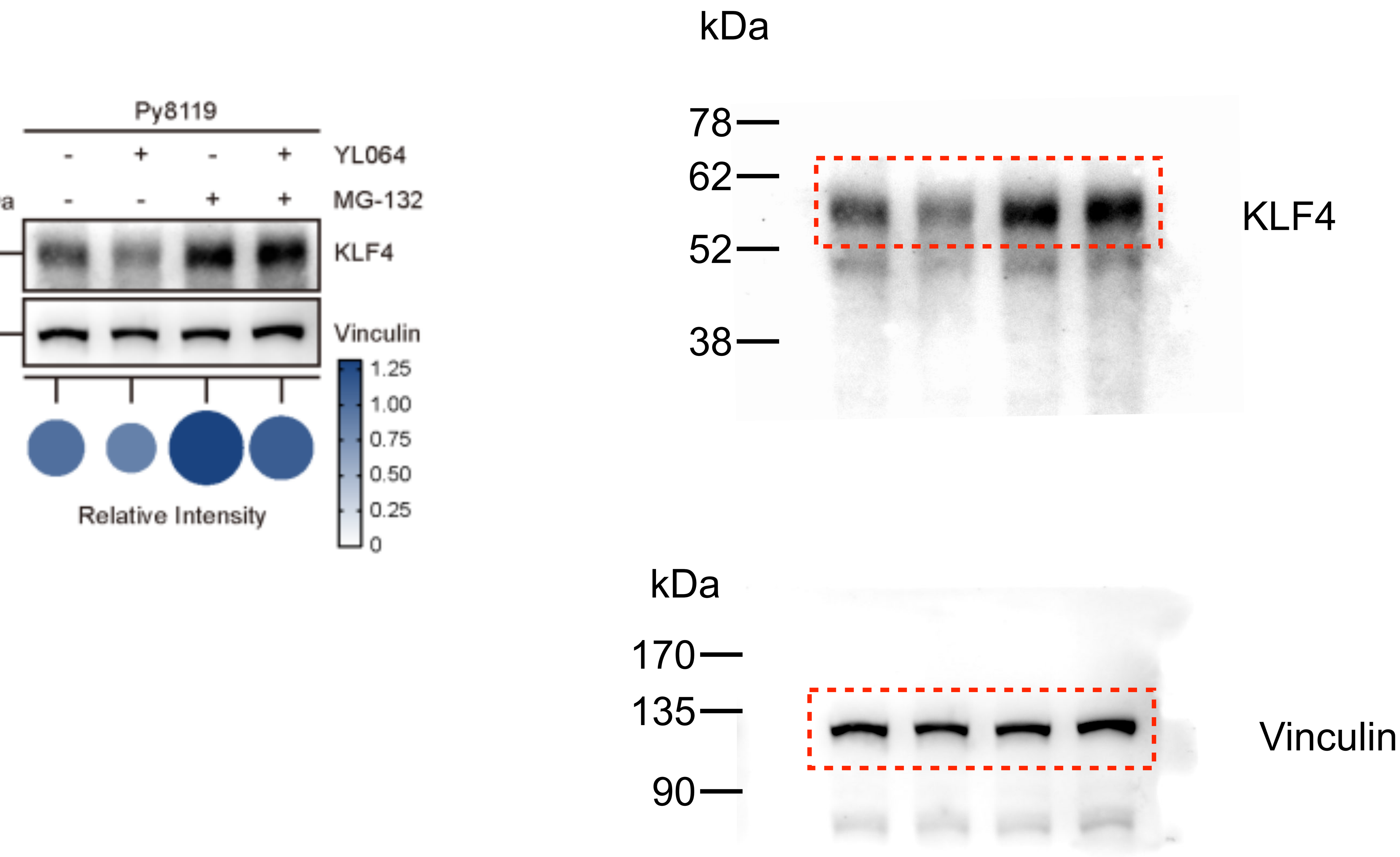

Py8119
YL064
MG-132
kDa
54
KLF4
117
Vinculin
1.25
1.00
0.75
0.50
0.25
0
Relative Intensity
kDa
78
62
52
38
KLF4
kDa
170
135
90
Vinculin

I

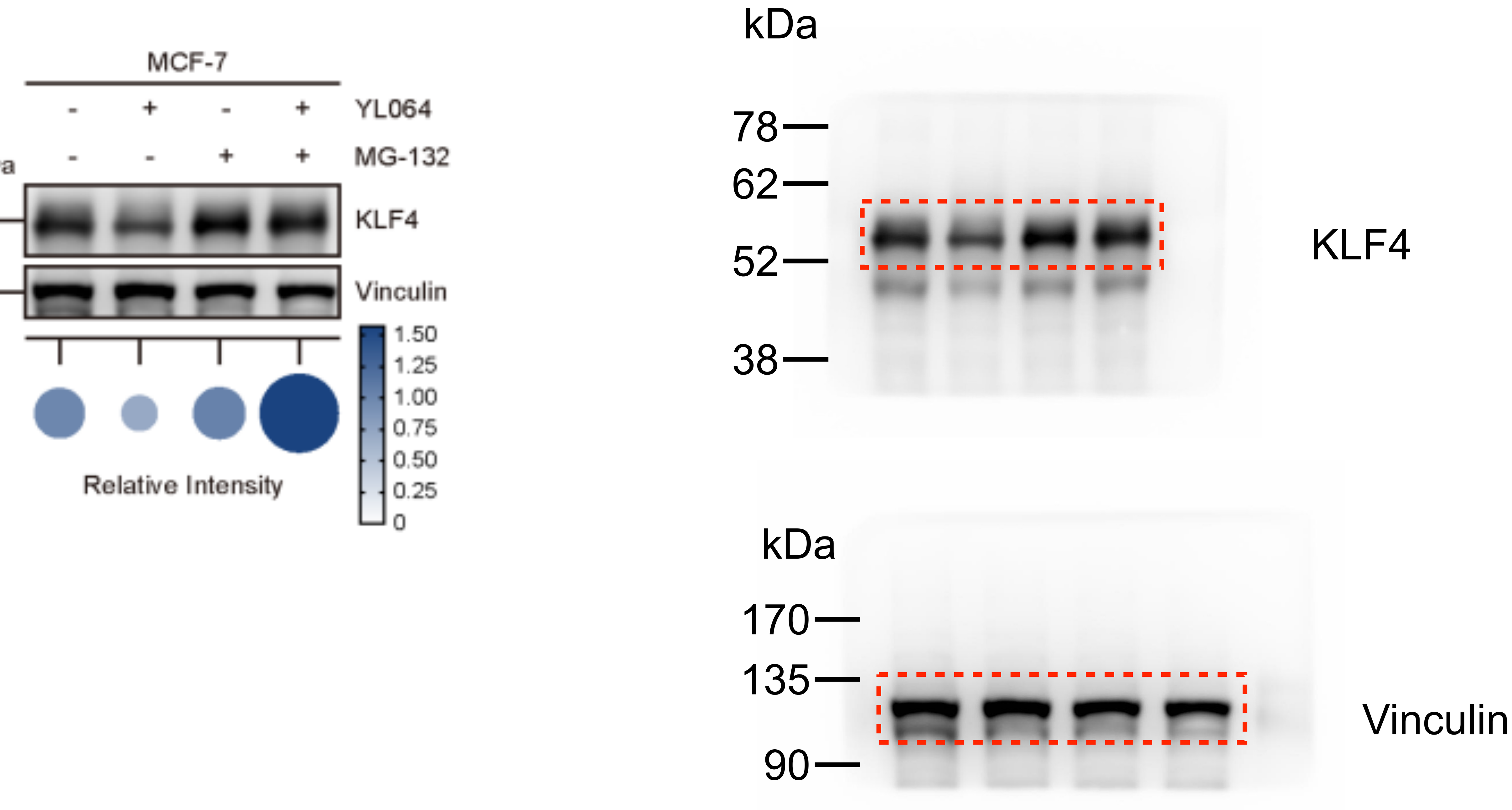

MCF-7
YL064
MG-132
kDa
54
KLF4
117
Vinculin
1.50
1.25
1.00
0.75
0.50
0.25
0
Relative Intensity
kDa
78
62
52
38
KLF4
kDa
170
135
90
Vinculin

J

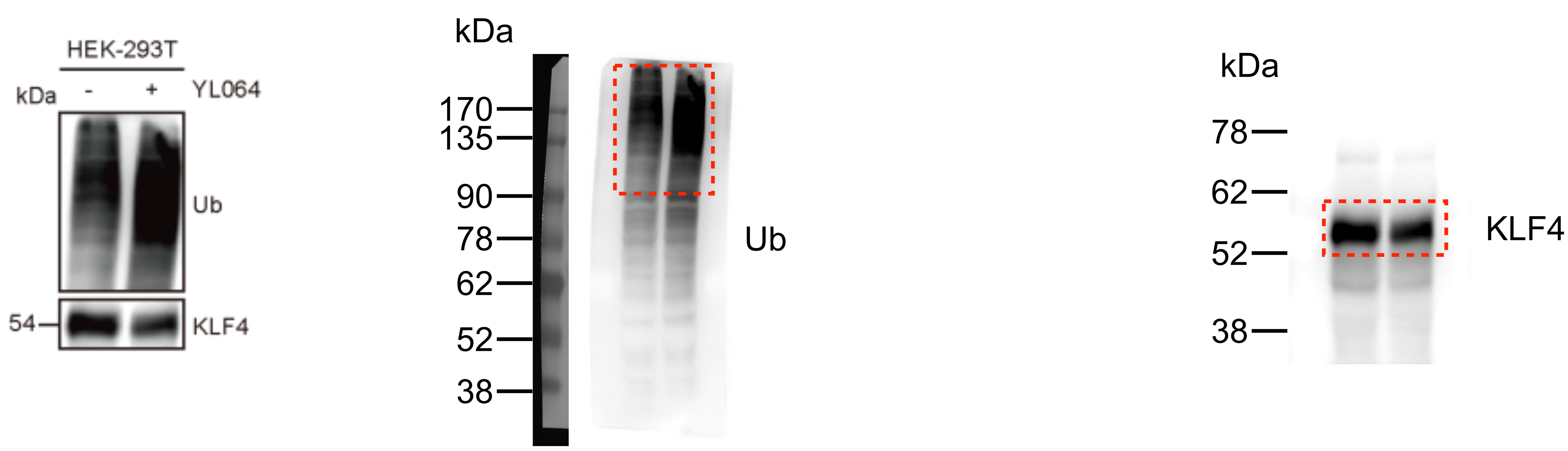

HEK-293T
kDa
YL064
Ub
54
KLF4
kDa
170
135
90
78
62
52
38
Ub
kDa
78
62
52
38
KLF4

Fig. 4C

	DMSO			YL064 20 μM		
Py8119	0.959784664	1.043909515	0.998075367	0.950829658	0.96900105	0.896567565
MCF-7	0.940487278	1.205913422	0.881720516	1.188535229	1.204178836	1.016794172

Fig. 5

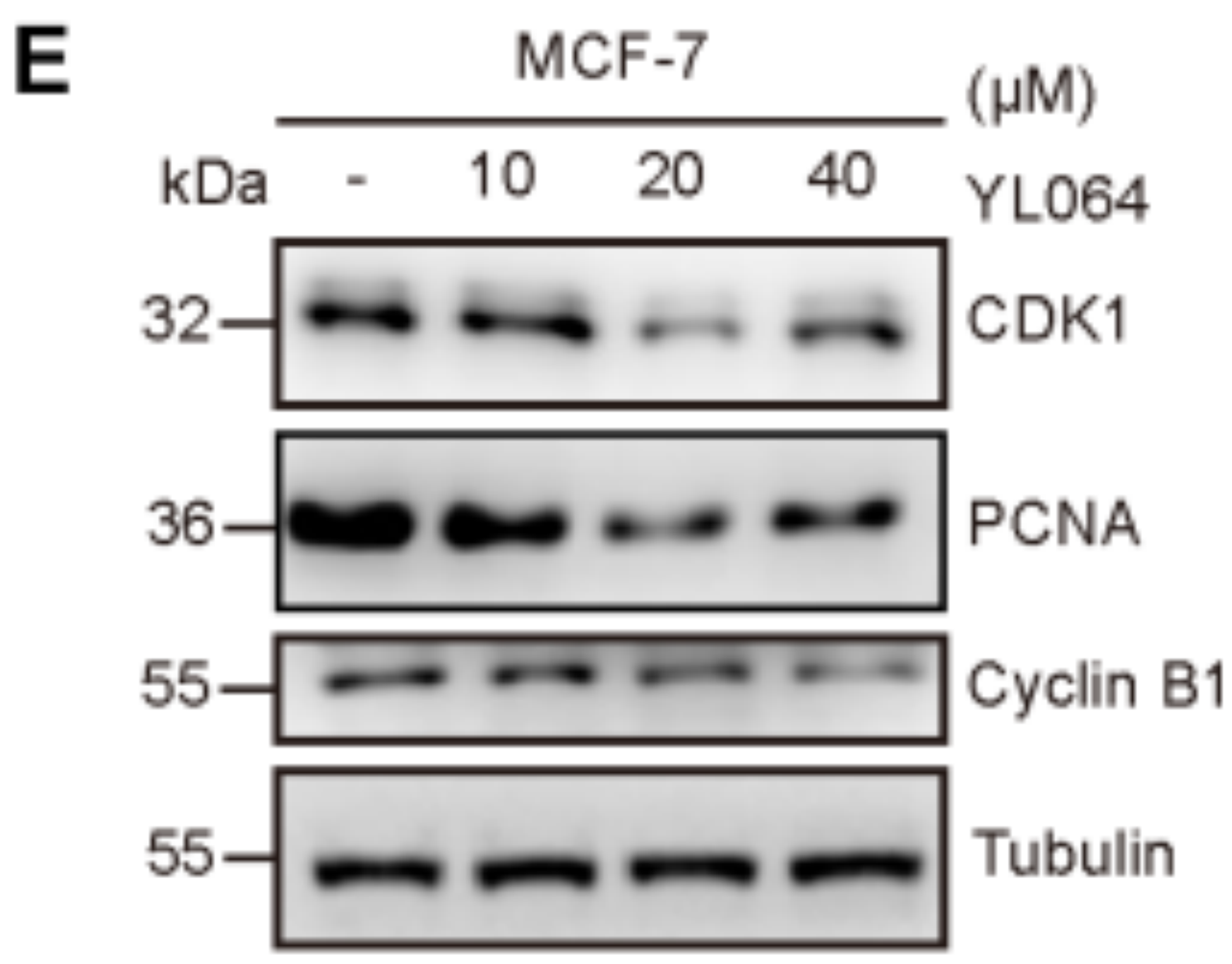

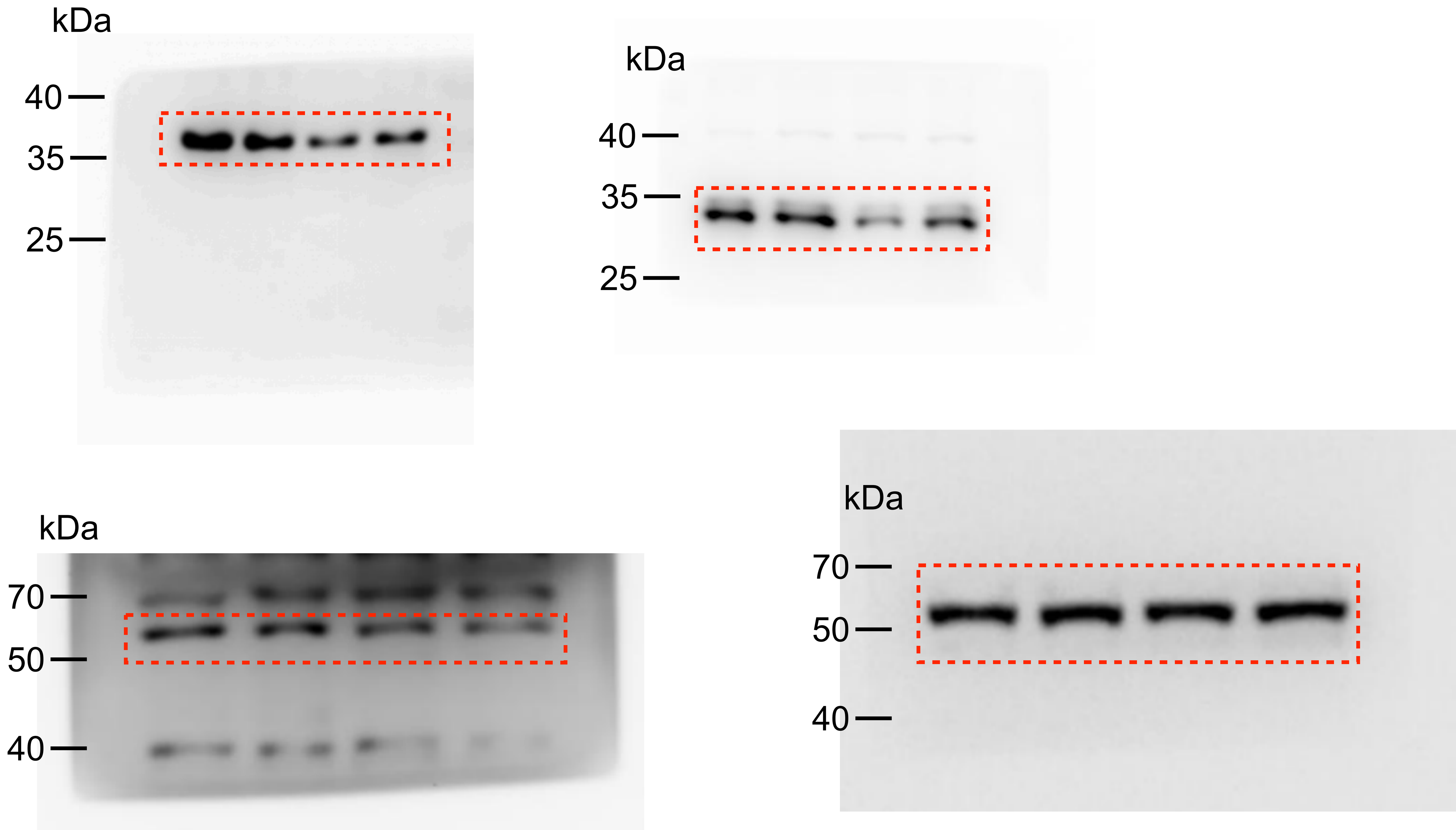

Fig. 6

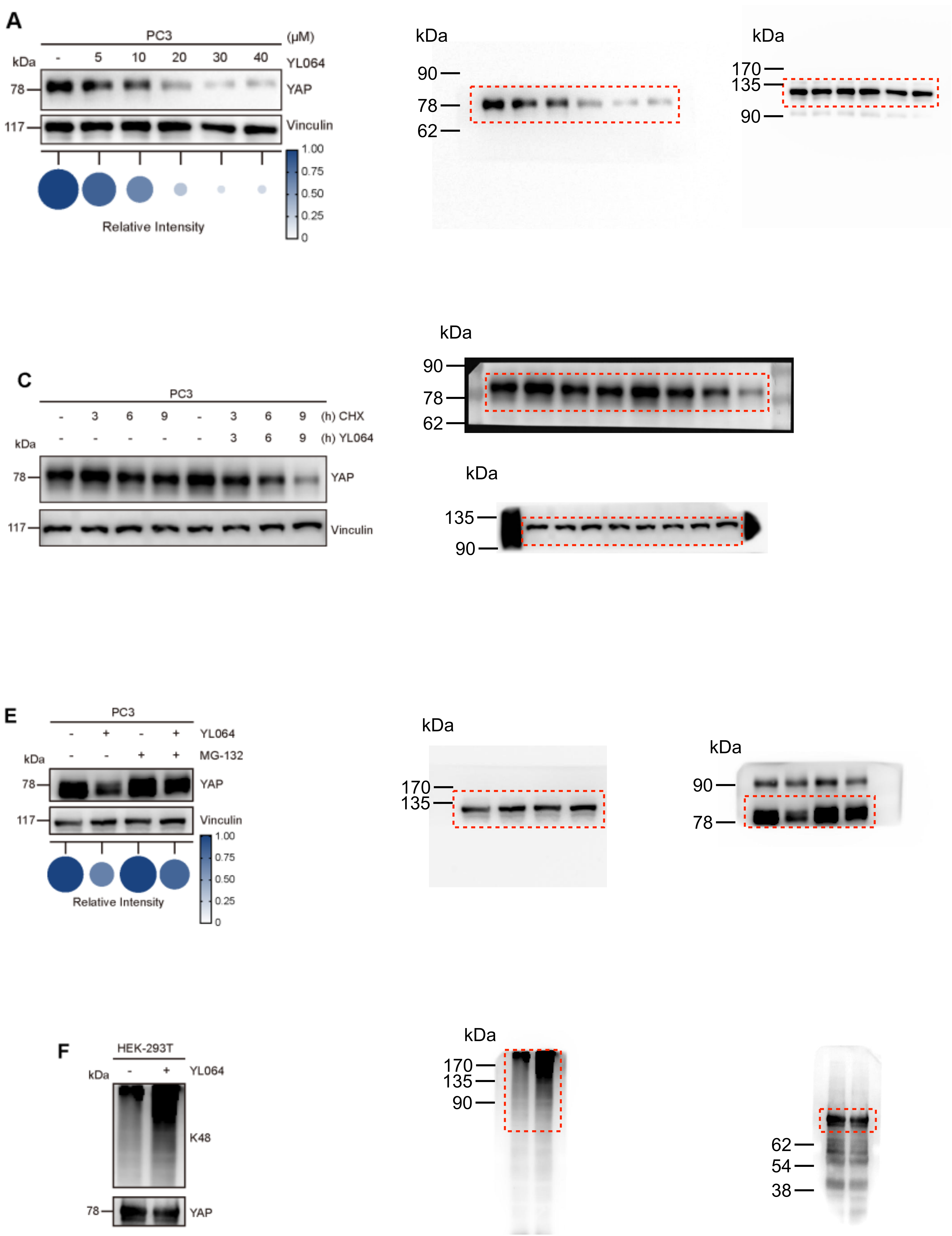

A
PC3
(μM)
kDa
- 5 10 20 30 40
YL064
78
YAP
117
Vinculin
1.00
0.75
0.50
0.25
0
Relative Intensity
kDa
90
78
62
kDa
170
135
90
C
PC3
- 3 6 9 - 3 6 9 (h) CHX
- - - - - 3 6 9 (h) YL064
kDa
78
YAP
117
Vinculin
kDa
90
78
62
kDa
135
90
E
PC3
- + - + YL064
- - + + MG-132
kDa
78
YAP
117
Vinculin
1.00
0.75
0.50
0.25
0
Relative Intensity
kDa
170
135
kDa
90
78
F
HEK-293T
kDa
- + YL064
K48
78
YAP
kDa
170
135
90
62
54
38

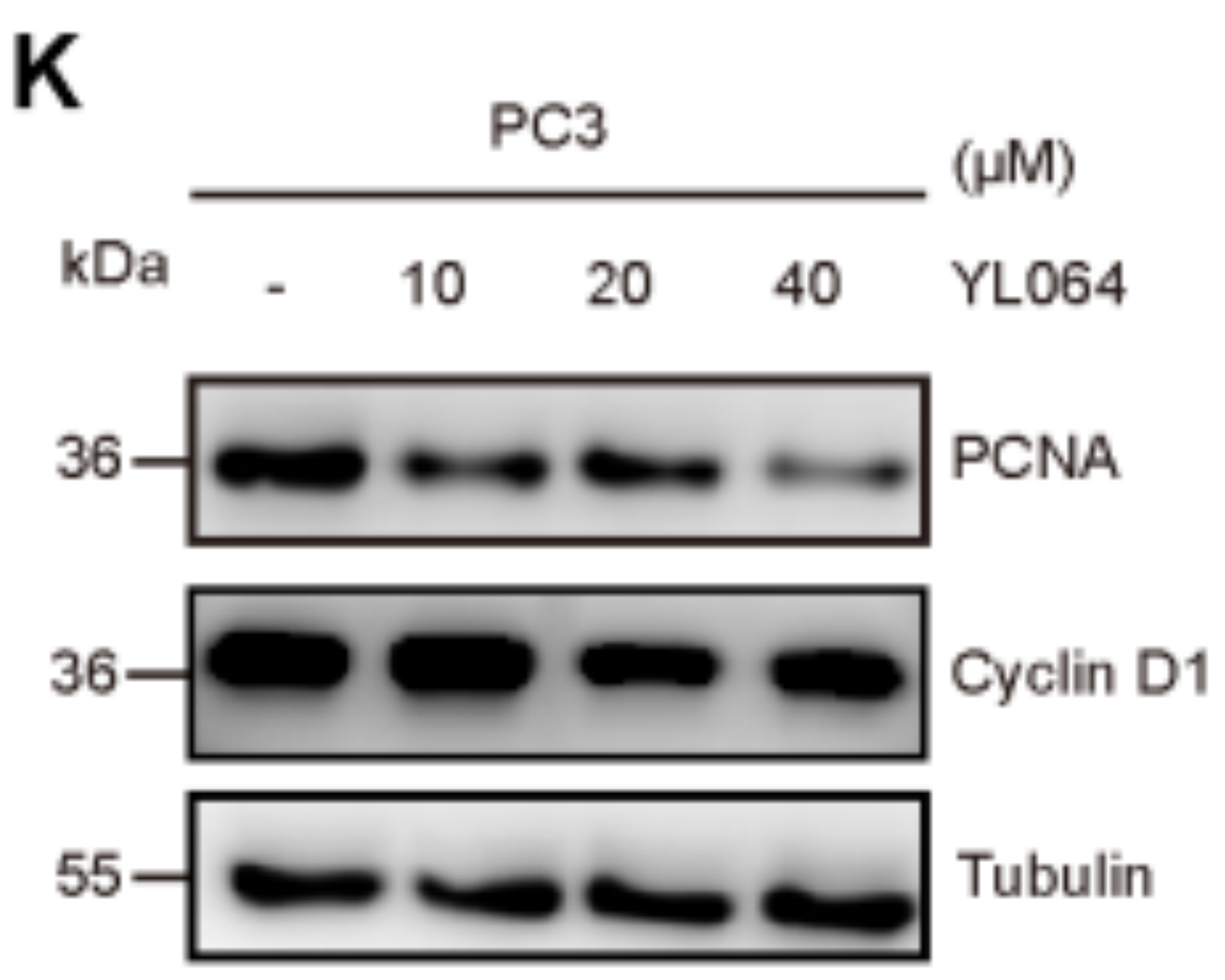

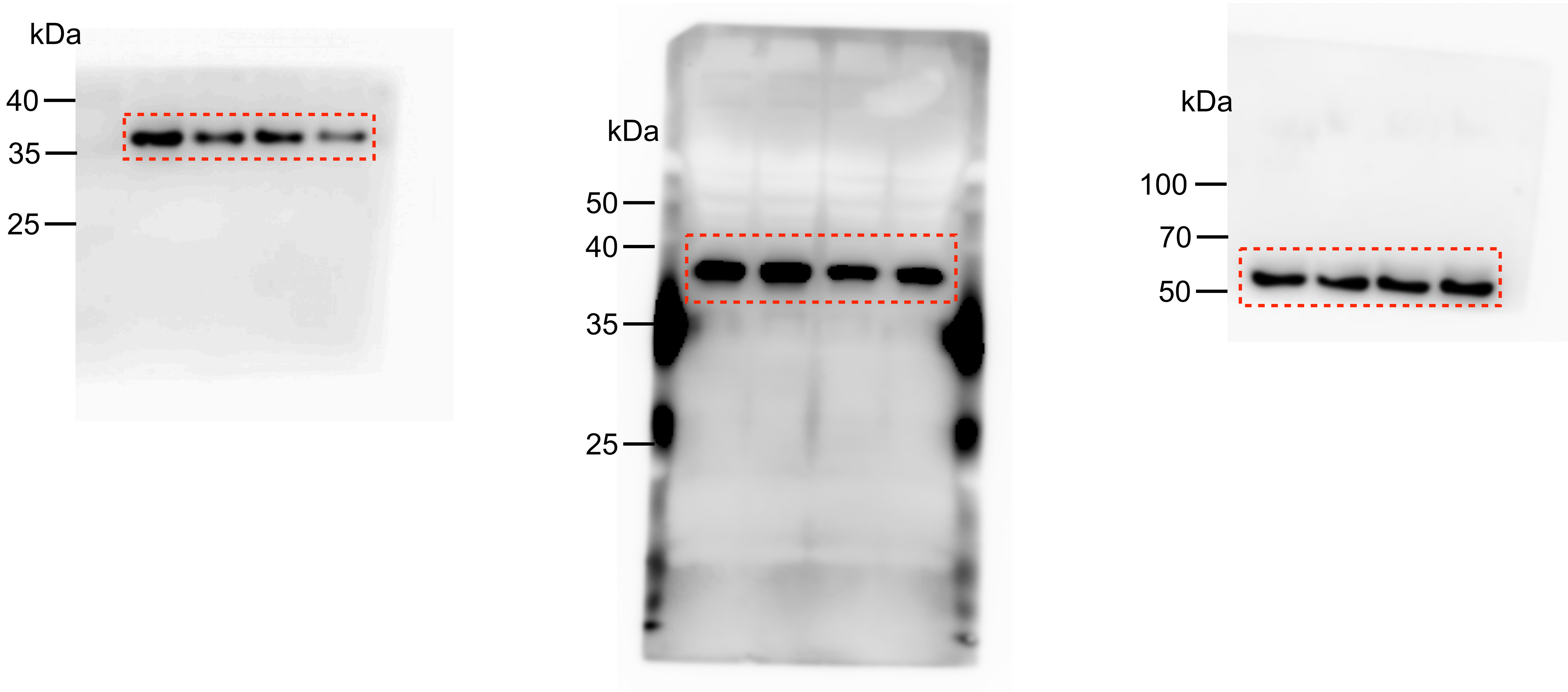

Fig. 6B

Relative YAP mRNA ratio						
	DMSO			YL064 20 μM		
YAP	0.9321735	1.1424336	0.9390145	0.9925261	1.0936886	1.2229513

Fig. 7

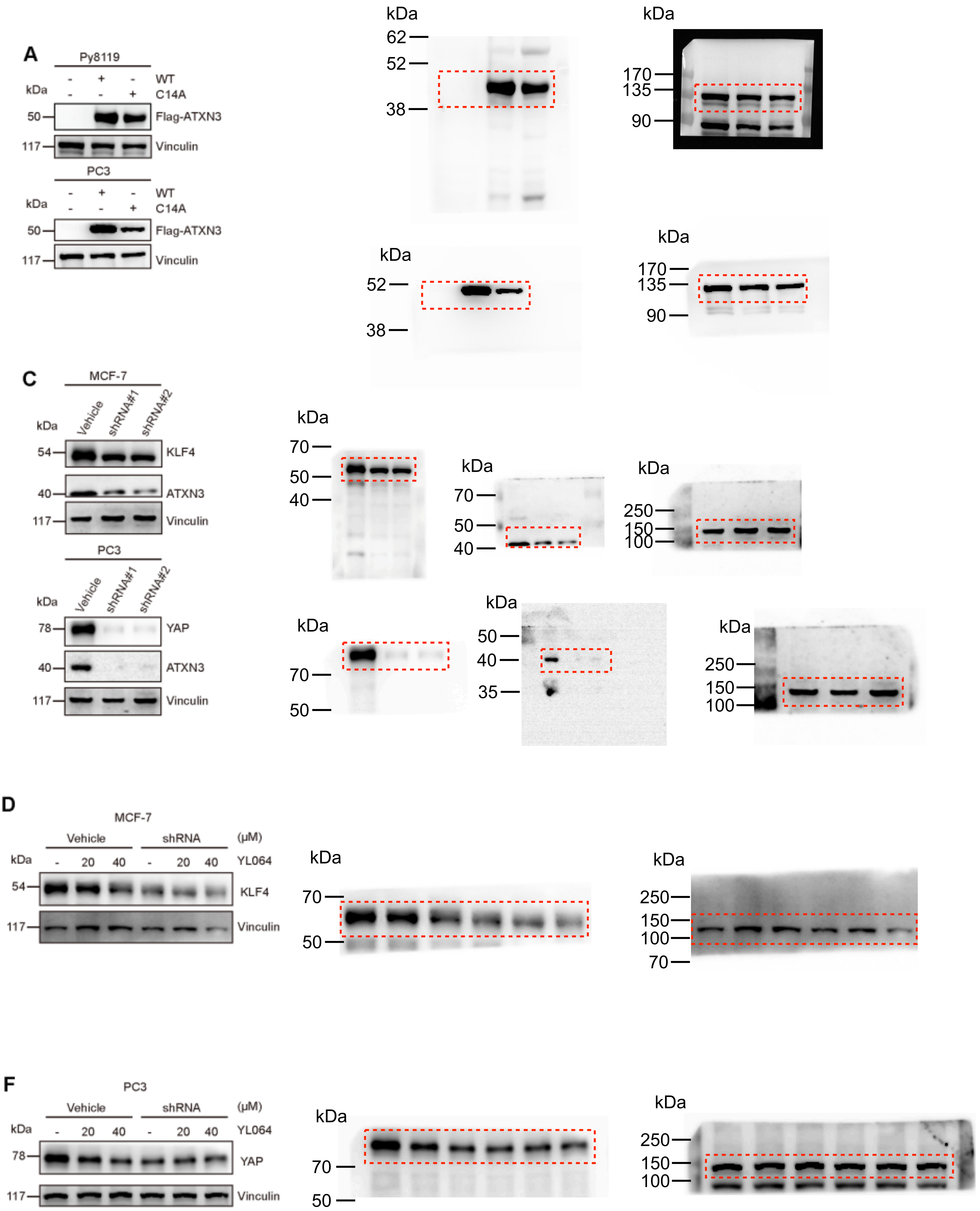
A
Py8119
WT
C14A
kDa
50
Flag-ATXN3
117
Vinculin
PC3
WT
C14A
kDa
50
Flag-ATXN3
117
Vinculin
kDa
62
52
38
kDa
170
135
90
kDa
52
38
kDa
170
135
90
C
MCF-7
kDa
Vehicle
shRNA#1
shRNA#2
54
KLF4
40
ATXN3
117
Vinculin
PC3
kDa
Vehicle
shRNA#1
shRNA#2
78
YAP
40
ATXN3
117
Vinculin
kDa
70
50
40
kDa
70
50
40
kDa
250
150
100
kDa
70
50
kDa
50
40
35
kDa
250
150
100
D
MCF-7
Vehicle
shRNA
(μM)
kDa
YL064
20
40
54
KLF4
117
Vinculin
kDa
70
50
kDa
250
150
100
70
F
PC3
Vehicle
shRNA
(μM)
kDa
YL064
20
40
78
YAP
117
Vinculin
kDa
70
50
kDa
250
150
100

Fig. 8BC Tumors weight

Vehicle	YL064	shRNA	shRNA+YL064
0.1353	0.0701	0.0285	0.0051
0.1539	0.0598	0.0278	0.0164
0.155	0.0788	0.0277	0.0128
0.206	0.0692	0.0277	0.0146

Fig. 8DE

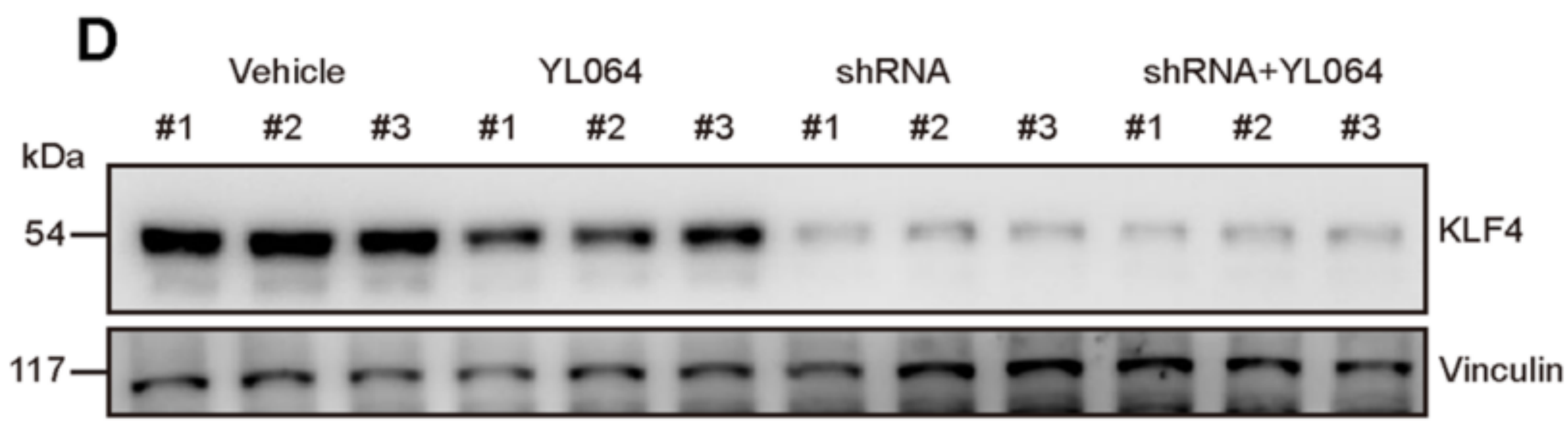

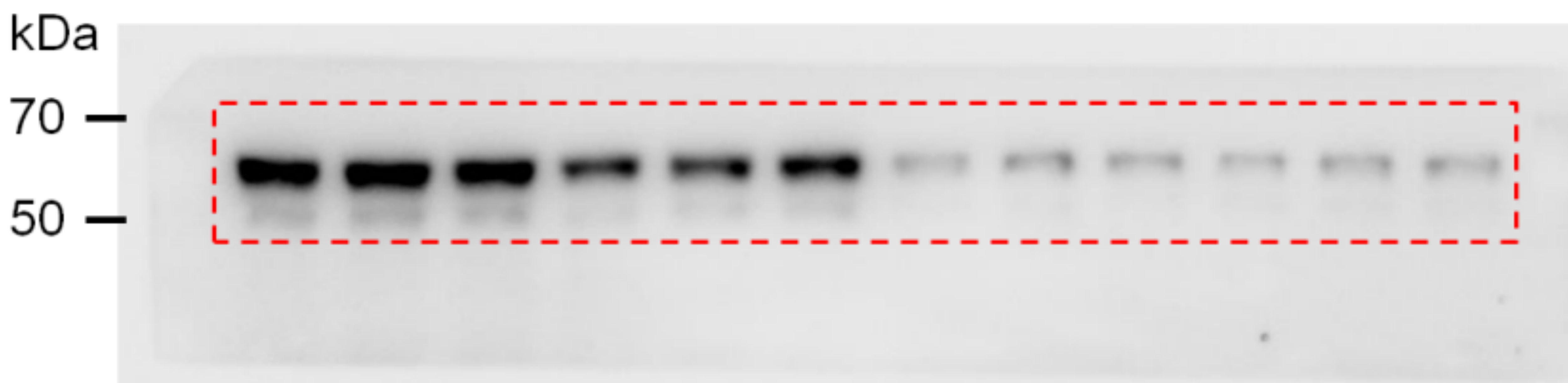

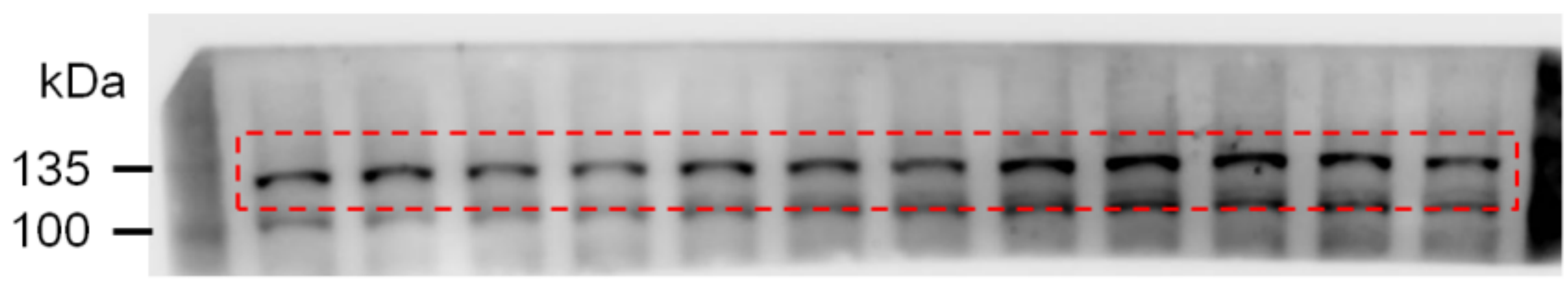

Fig. 8F

	Relative KLF4 mRNA ratio					
	Vehicle			YL064		
KLF4	0.982139595	0.942131274	1.080725402	1.085981856	0.881480158	1.098854218

Fig. S2A

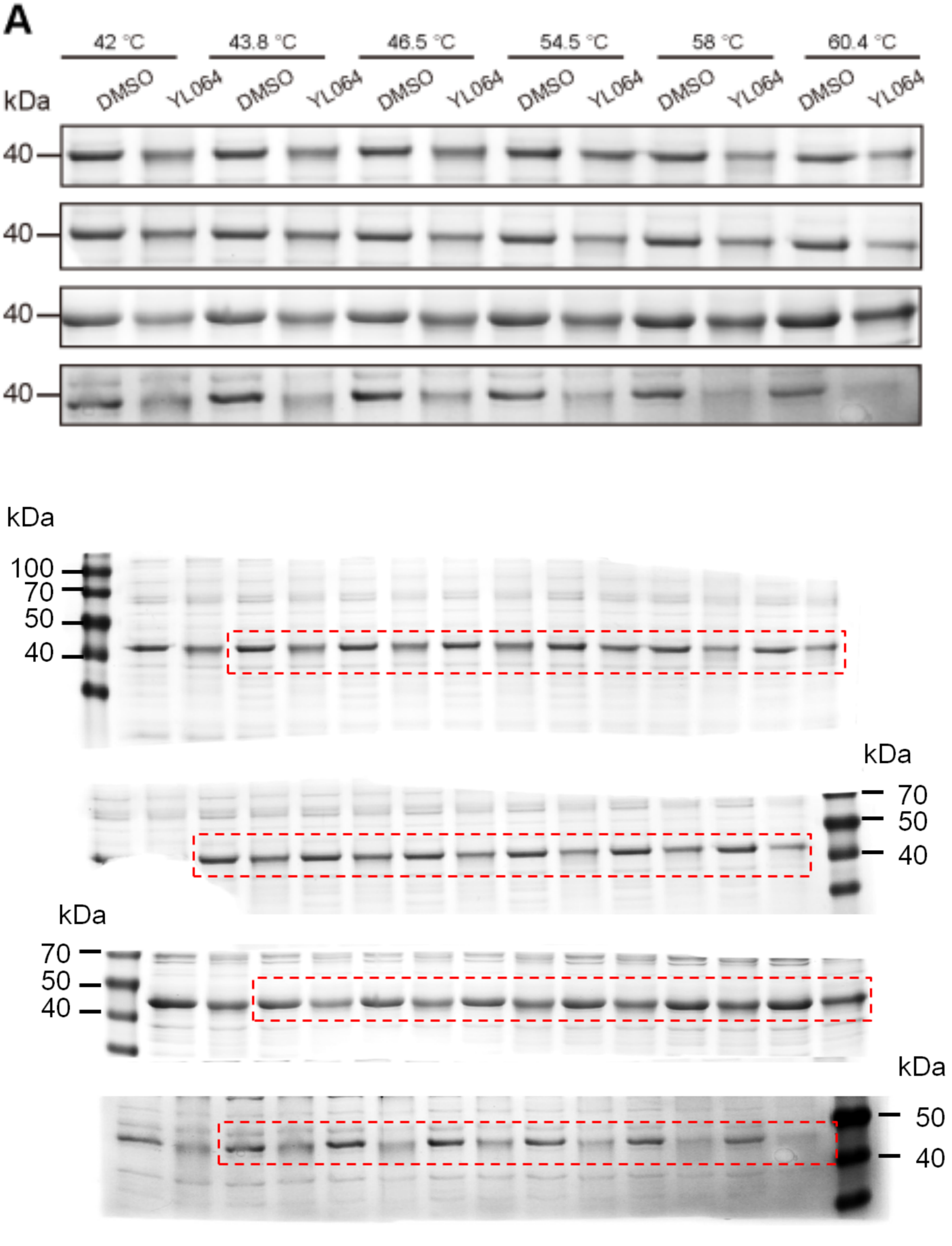
A
42 °C
43.8 °C
46.5 °C
54.5 °C
58 °C
60.4 °C
kDa
DMSO
YL064
40
kDa
100
70
50
40
kDa
70
50
40
kDa
70
50
40
kDa
50
40

Fig. S2B

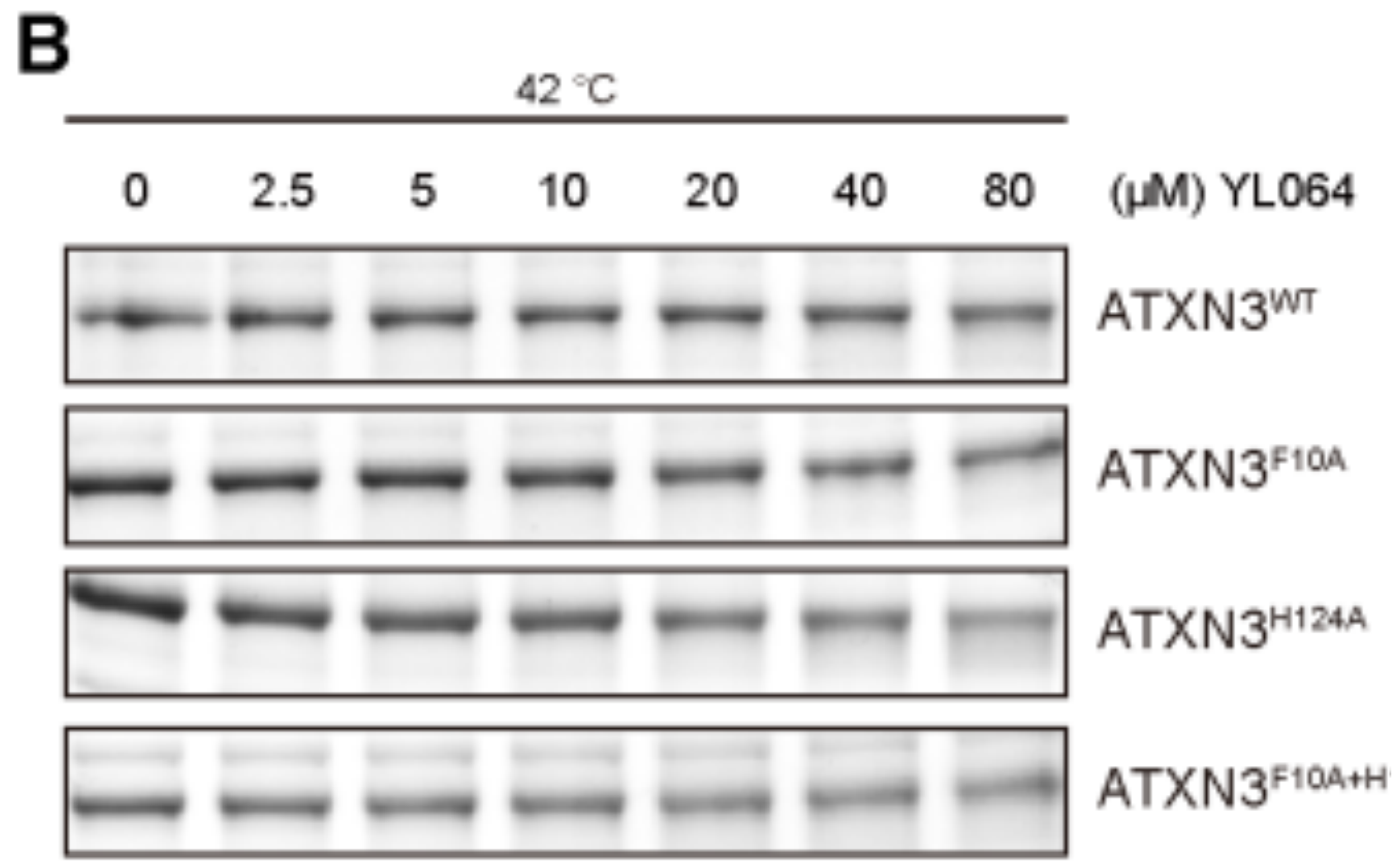

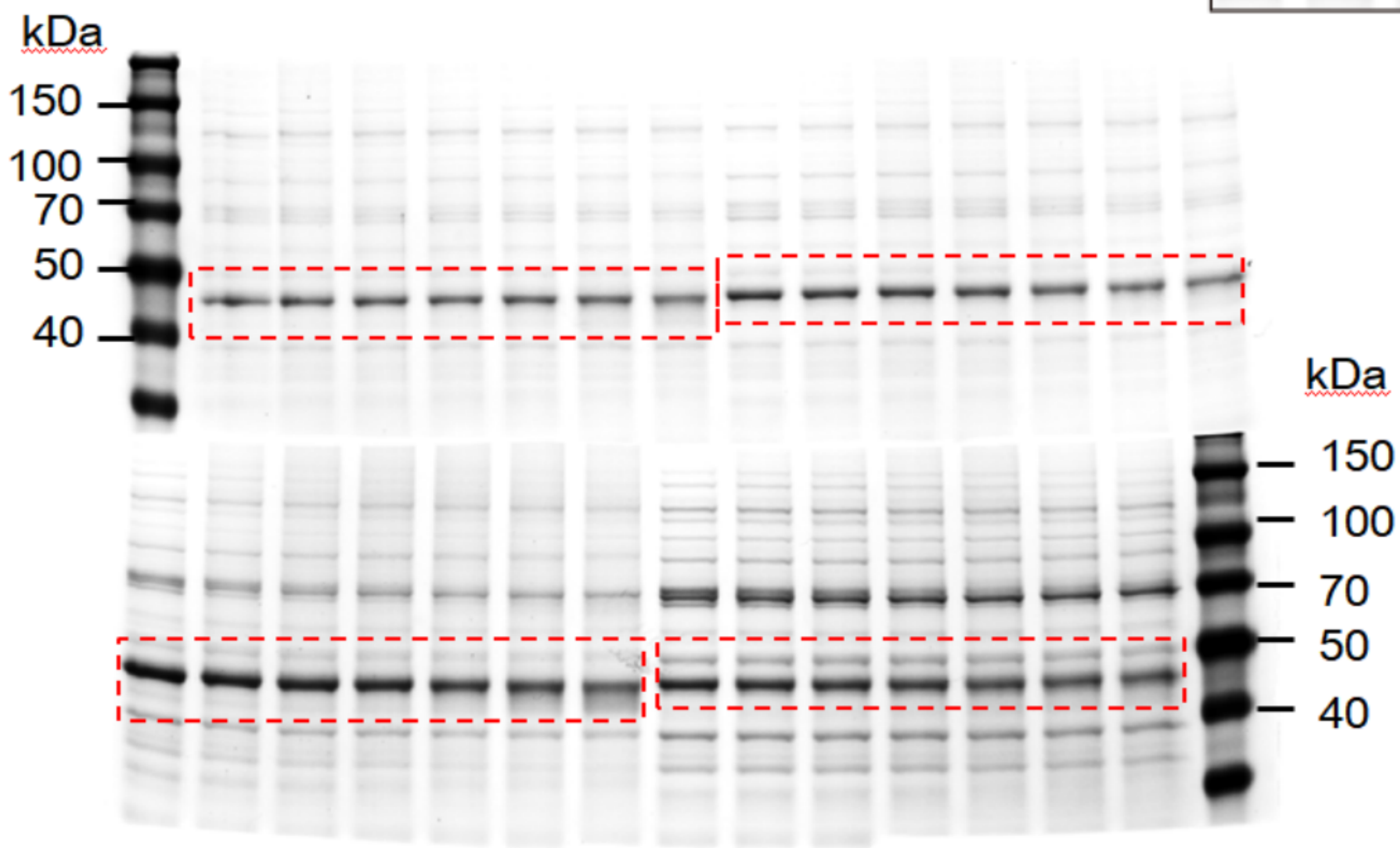

Fig. S2C

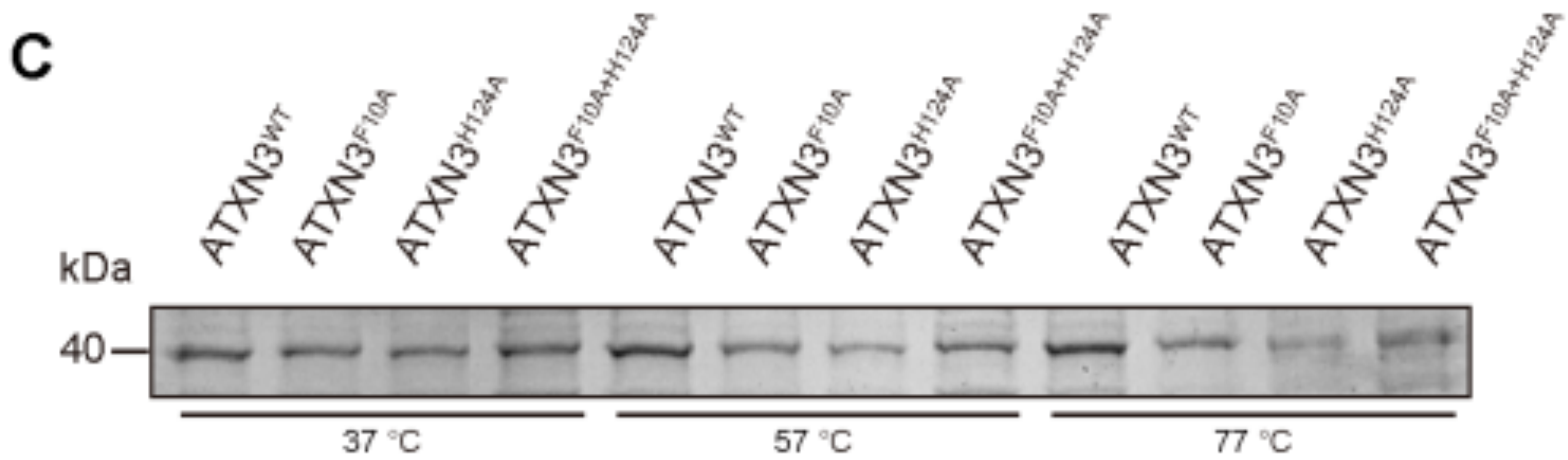

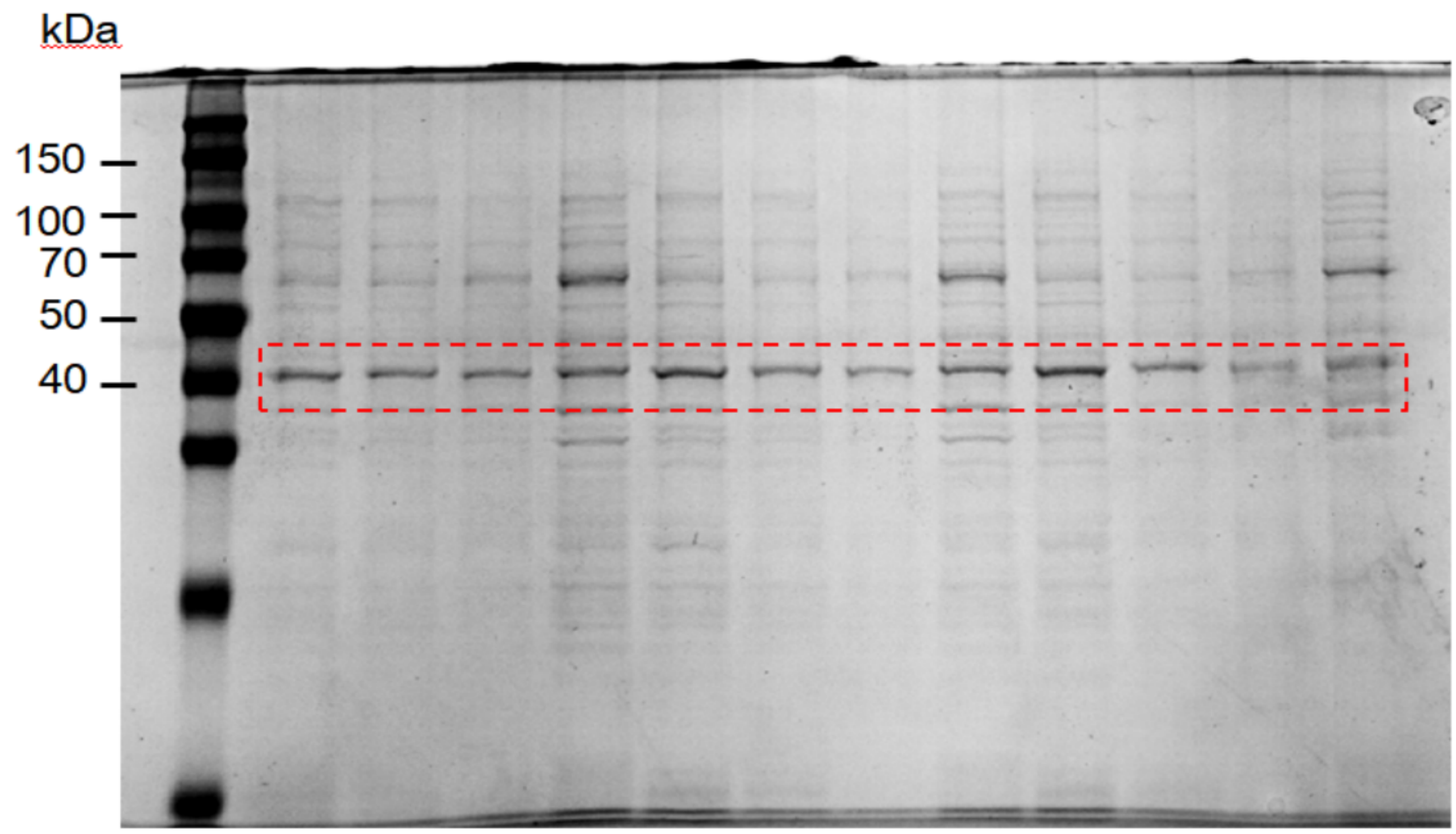

Fig. S3A

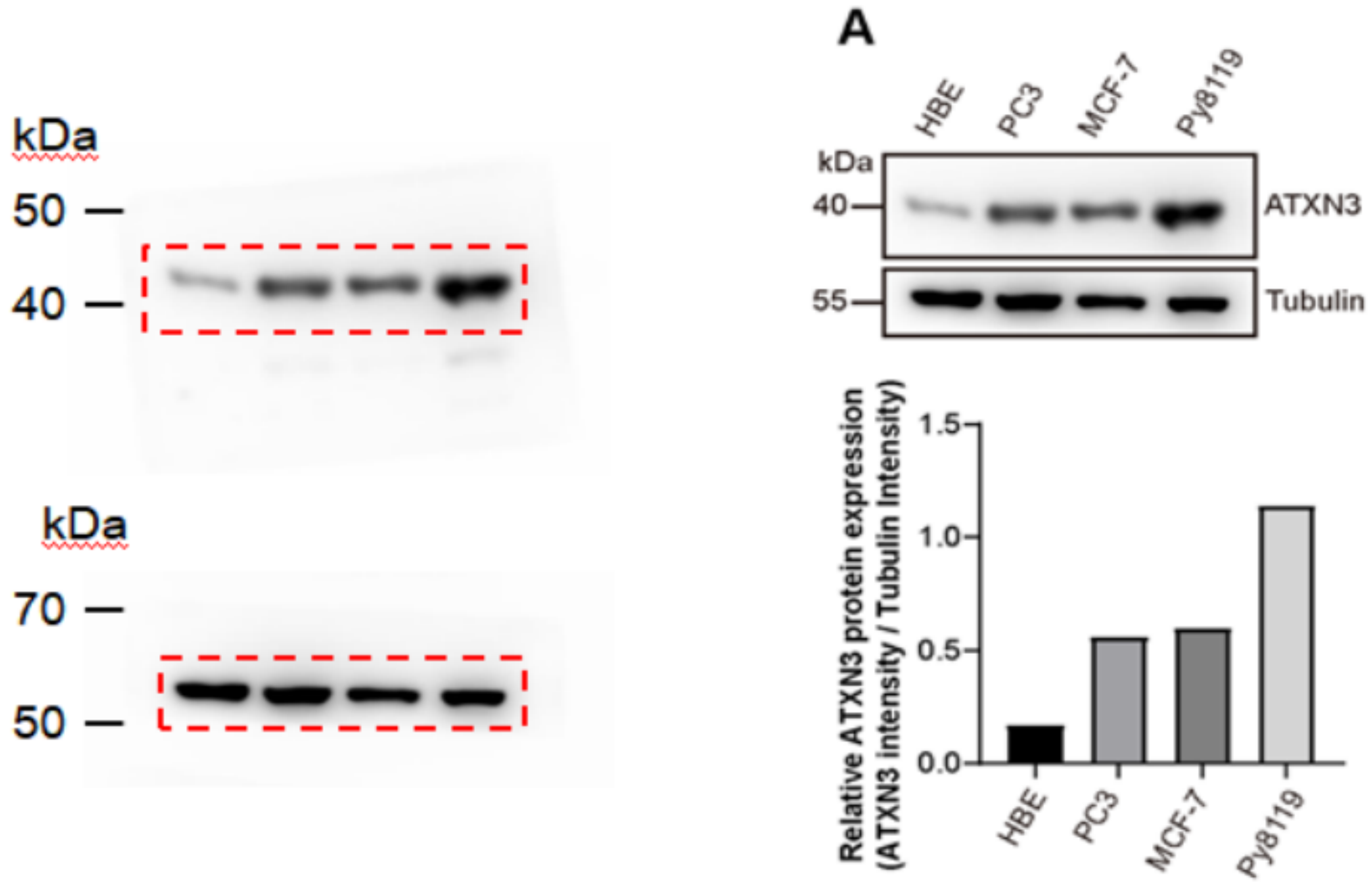

Fig. S4B

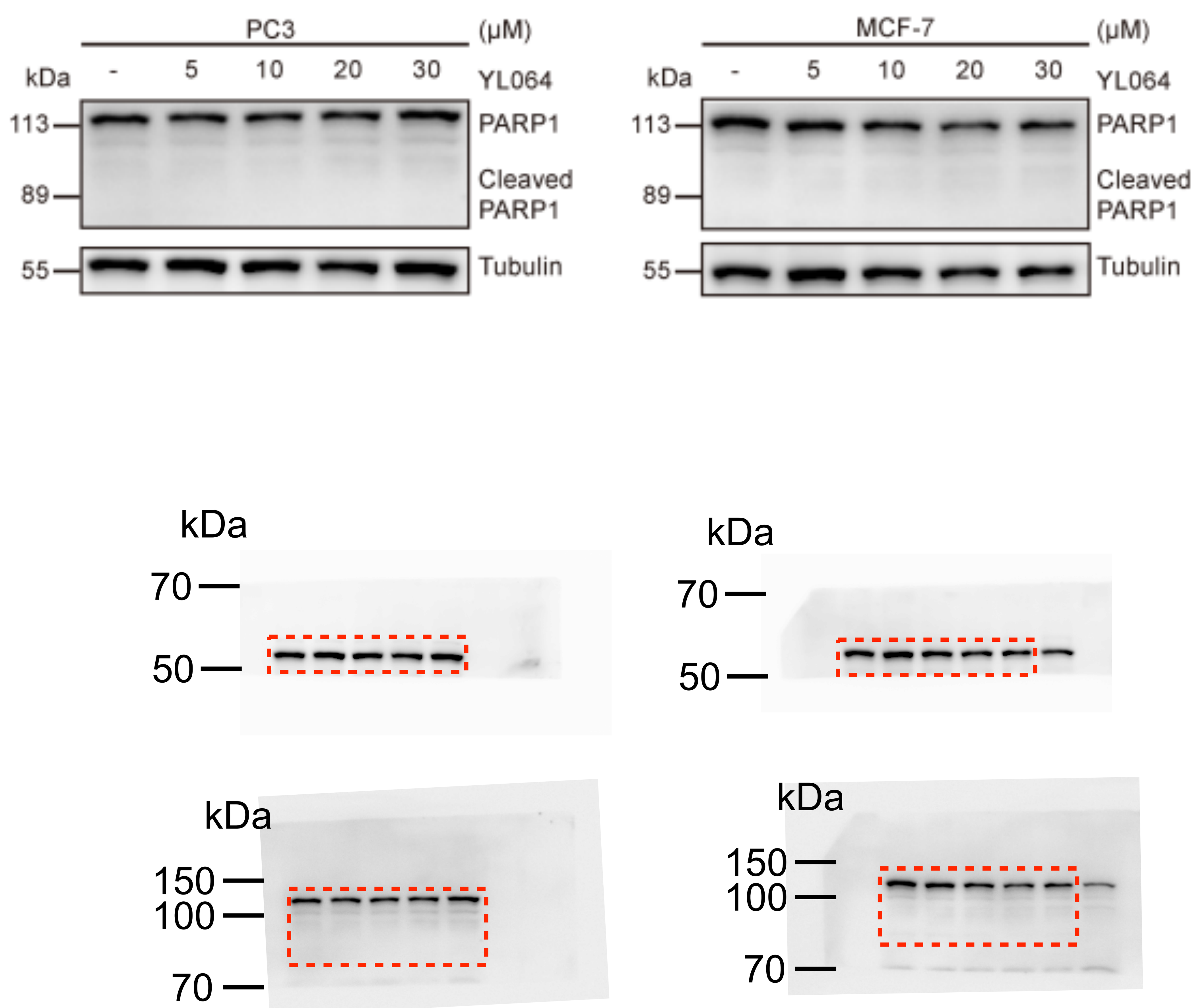

Supplement: Supplementary file 1 [file 41419_2026_9171_MOESM2_ESM.pdf]
