# Supplementary material for: Identification of YL064 as a novel inhibitor of ATXN3 to suppress tumor growth and metastasis by promoting degradation of oncogenic substrates
Source: Cell Death Dis. 2026 Aug 6;17(1):934. doi: 10.1038/s41419-026-09171-5 (PMC13728240; doi:10.1038/s41419-026-09171-5)
Supplement: Supplementary file 2 [file 41419_2026_9171_MOESM1_ESM.docx]

***Supplementary Materials***

**Identification of YL064 as a novel inhibitor of ATXN3 to suppress tumor growth and metastasis by promoting degradation of oncogenic substrates**

**Supplementary Table S1:** Primer sequences for qPCR.

| Species | Sequence (5’ to 3’) | | Primer name |
| --- | --- | --- | --- |
| Human | Forward | TCCACGAGAAACAAGAAGGCT | *ATXN3* |
|  | Reverse | TCCTCTCCTCCTCATCCAGC |  |
| Human | Forward | GGACACACGGGATGATGCTC | *KLF4* |
|  | Reverse | TTCTCACCTGTGTGGGTTCG |  |
| Human | Forward | TAGCCCTGCGTAGCCAGTTA | *YAP* |
|  | Reverse | TCATGCTTAGTCCACTGTCTGT |  |
| Human | Forward | CCTTCCTTCCTGGGCATGG | *ACTB* |
|  | Reverse | TCTTCATTGTGCTGGGTGCC |  |
| Mouse | Forward | TCCACGAGAAACAAGAAGGCT | *Atxn3* |
|  | Reverse | TCCTCTCCTCCTCATCCAGC |  |
| Mouse | Forward | GAAGGGAGAAGACACTGCGT | *Klf4* |
|  | Reverse | GTCTAGGTCCAGGAGGTCGT |  |
| Mouse | Forward | TATAAAACCCGGCGGCGCA | *Actb* |
|  | Reverse | TCATCCATGGCGAACTGGTG |  |

**Supplementary Table S2: The sequences of *ATXN3* small hairpin RNAs (shRNAs)**.

| Species | shRNAs Sequences | |
| --- | --- | --- |
| Human | shRNA #1 | GGAAGAGACGAGAAGCCTACT |
|  | shRNA #2 | CAGGAATGTTAGACGAAGATG |
| Mouse | shRNA #1 | CAGGTGAACTCCATGCTAA |
|  | shRNA #2 | CAGATTGGATCAGTAGTAT |

**Supplementary Table S3:** The results of the ATXN3 activity inhibitory rate of Sinomenine and its derivatives.

| Compounds | Structural formula | Inhibitory rate |
| --- | --- | --- |
| Sinomenine |  | 81.08% |
| YL064 |  | 85.90% |
| Derivative 1 |  | 84.13% |
| Derivative 2 |  | 60.47% |
| Derivative 3 |  | 30.26% |
| Derivative 4 |  | 39.14% |
| Derivative 5 |  | 68.73% |
| Derivative 6 |  | 84.05% |
| Derivative 7 |  | 68.08% |
| Derivative 8 |  | 60.76% |
| Derivative 9 |  | 67.76% |
| Derivative 10 |  | 69.88% |
| Derivative 11 |  | 57.91% |
| Derivative 12 |  | 77.76% |
| Derivative 13 |  | 83.67% |
| Derivative 14 |  | 47.48% |
| Derivative 15 |  | 85.57% |
| Derivative 16 |  | 80.11% |
| Derivative 17 |  | 47.74% |
| Derivative 18 |  | 51.88% |
| Derivative 19 |  | 59.47% |
| Derivative 20 |  | 53.86% |
| Derivative 21 |  | 55.15% |
| Derivative 22 |  | 61.58% |
| Derivative 23 |  | 51.00% |
| Derivative 24 |  | 44.62% |
| Derivative 25 |  | 64.61% |

**Figure S1. Identification of YL064 as a potential inhibitor of ATXN3.**

Dose-response curves of four additional potential ATXN3 inhibitors (CDDO-Me, cinobufagin, adenanthin, and Oridonin). ATXN3 activity was quantified using the Ub-AMC assay after incubation with gradient concentrations of drugs.

R.U. denotes relative fluorescence units.

**Figure S2. Effect of ATXN3 mutation on its thermal stability and interaction with YL064.**

**A** TSA results showing the interaction between purified ATXN3 proteins (WT, F10A, H124A, and double mutant) and YL064 (100 μM) at different temperatures. **B** TSA showing the interaction between purified ATXN3 proteins (WT, F10A, H124A, and F10A/H124A double mutant) and different concentrations of YL064 at 42 °C. **C** TSA comparing the intrinsic thermal stability of purified ATXN3 proteins (WT, F10A, H124A, and F10A/H124A double mutant) without treatment.

**Figure S3. ATXN3 is a critical target in breast cancer and prostate cancer.**

**A** Western blot analysis and relative quantification of ATXN3 protein expression in the normal epithelial cell line HBE and cancer cell lines used in this study. ATXN3 was highly expressed in the MCF-7, Py8119, and PC-3 cell lines compared with the HBE cell line. **B** ATXN3 expression in different breast and prostate cancer cell lines. The data were obtained from the Human Protein Atlas ([https://www.proteinatlas.org/).](https://www.proteinatlas.org/.)

**Figure S4. ATXN3 depletion and YL064 do not induce apoptosis.**

**A** Flow cytometric analysis of PI staining showed no significant apoptosis in parental MCF-7 or *ATXN3*-knockdown MCF-7 cells treated with or without YL064. **B** Immunoblot analysis showed no detectable PARP1 cleavage after YL064 treatment in MCF-7 and PC-3 cells.
